# Estimation of Area Deprivation Index Rankings in South Dakota Using Spatiotemporal Change of Support Method

**DOI:** 10.1080/2330443x.2026.2704953

**Published:** 2026-09-09

**Authors:** Annika Spors, Semhar Michael, Patti Brooks, Brandon Varilek, Surachat Ngorsuraches, Hossein Moradi Rekabdarkolaee

**Affiliations:** a Department of Mathematics and Statistics, South Dakota State University, Brookings, SD; b College of Business and Information Systems, Dakota State University, Madison, SD; c College of Nursing, University of Nebraska Medical Center, Omaha, NE; d Department of Health Outcomes Research and Policy, Auburn University, Auburn, AL; e Department of Business Analytics, Economics, and Information Systems, Bowling Green State University, Bowling Green, OH

**Keywords:** Bayesian inference, Gibbs sampling, Social determinant of health, Spatial correlation, Variance estimation

## Abstract

Social determinants of health (SDOH) are estimated to drive nearly 80% of health outcomes, making the accurate measurement of neighborhood disadvantage essential for equitable public health policy and resource allocation. The Area Deprivation Index (ADI) serves as a critical multidimensional proxy for SDOH; however, its utility is frequently hindered by a “change of support” problem, where the geographic resolution of available data (census block groups) does not align with the scales required for policy implementation, such as ZIP Code Tabulation Areas (ZCTAs). This mismatch can lead to biased inferences and the misidentification of high-risk communities. This study employs a spatiotemporal change of support (STCOS) model to bridge these scales, specifically addressing the challenge that ADI rankings lack the known variance measures required for robust statistical modeling. We propose and evaluate several variance estimation techniques, including multiple linear regression and finite mixture models, to generate reliable, uncertainty-aware ADI estimates for South Dakota at the ZCTA level. Our findings demonstrate that these refined estimates can successfully identify areas of elevated social risk, even in regions with missing data. By providing a statistically rigorous framework for harmonizing mismatched health data, this research offers a vital tool for decision-makers to support health equity initiatives, such as the Centers for Medicare & Medicaid Services Accountable Care Organization Realizing Equity, Access, and Community Health Model, known as the ACO REACH Model, and to ensure that interventions and resources are precisely directed toward the most underserved populations.

## 1. Introduction

Rising healthcare expenditures in the United States continue to outpace inflation and the growth of wages, yet the health of the population appears to be deteriorating (Hayes and Delk 2018; Gunja, Gumas, and Williams 2023). A growing body of evidence suggests that medical care accounts for only a small proportion of overall health outcomes, while the majority—often estimated at roughly 80%—is driven by non-medical socioeconomic factors, environmental factors, and health-related behaviors (Magnan 2017). These are broadly called social determinants (or drivers) of health. Social determinants of health (SDOH) comprise factors across five domains that can affect people’s health (Office of Disease Prevention and Health Promotion 2023; World Health Organization 2023). The five domains include: (a) economic stability, (b) education access and quality, (c) healthcare access and quality, (d) neighborhood and built environment, and (e) social and community context (Agency for Healthcare Research and Quality 2023).

Capturing the broad implications of SDOH can be challenging, but indices are available that provide discrete, area-level measures of SDOH impact. The Area Deprivation Index (ADI) is an example of a multidimensional index that assigns a numeric value of 1–100 to census-tract units based on 17 material and socioeconomic variables gathered from the U.S. (Knighton et al. 2016; Durfey et al. 2019; Johnson et al. 2021). Because its component variables span economic stability (i.e., income and employment), education access and quality, housing and neighborhood conditions, and social context, ADI is widely used in health services research as an area-level proxy for SDOH and has been associated with patterns of healthcare utilization and spending. Examples include using the ADI scores to identify deprived needs when developing community health needs assessments, identifying high-risk patients for care management intervention, and assessing the impact of deprivation on treatment of patients with hypertension (Kind et al. 2014; Knighton et al. 2016; Hu, Kind, and Nerenz 2018). Similarly, Johnson et al. (2021) used ADI to measure neighborhood deprivation when assessing the risk of hospital readmissions and mortality among patients with heart failure, myocardial ischemia, and atrial fibrillation. Thus, as a descriptive proxy for SDOH, the ADI can help identify neighborhoods with elevated social risk, guide proactive and preventive interventions, and support more equitable allocation of health care and social service resources (Maroko et al. 2016; Kind and Buckingham 2018).

ADI rankings (as it can be seen in Figure 1) are correlated over space and time, making them well suited for analysis using spatiotemporal methods (Cressie and Wikle 2011). One of the challenges of analyzing spatiotemporal data is that the spatial support of observed data does not always align with the desired analytic support. This issue arises, for example, when applying ADI rankings to retrospective cohort studies in which geospatial resolutions differ, such as when secondary data sources are available only at the 5-digit ZIP code level rather than at the census-tract or block level. This situation is known as the change-of-support (COS) problem (Bradley, Wikle, and Holan 2015). Given the importance of accurately linking area-level deprivation measures to health outcomes and utilization, there exists an extensive body of literature addressing the COS problem (Gotway and Young 2002; Cressie and Wikle 2011; Banerjee, Carlin, and Gelfand 2015).

A standard approach to addressing spatial change-of-support (COS) is areal-weighted interpolation, in which values are estimated in proportion to the areas of overlap between source and target spatial supports. While this method is straightforward to implement, it is limited to purely spatial contexts and does not provide measures of uncertainty in the resulting estimates (Bradley, Wikle, and Holan 2015). To address these limitations, the spatiotemporal change-of-support (STCOS) method has been proposed (Bradley, Wikle, and Holan 2015; Raim et al. 2021). This framework explicitly accounts for differences in both geography and time and provides associated measures of uncertainty. ADI rankings, however, are calculated at the census block group level and are typically reported without variance estimates. In contrast, the STCOS model requires that the variance associated with each observed data value be specified, as these variances can substantially influence the accuracy of estimates at the target support. Multiple approaches exist for estimating these variances. In this study, we employed several variance estimation techniques, including methods based on k-nearest neighbors, k-means clustering, hierarchical clustering with spatial constraints, multiple linear regression, and finite mixture models.

The rest of this article is organized as follows. Section 2 gives further details on the ADI rankings. Section 3 provides a brief review of the STCOS model and the methods used for the variance estimation. Section 4 outlines how the STCOS model was tuned. Section 5 demonstrates the performance of the STCOS model by estimating the ADI rankings in the state of South Dakota at the ZIP Code Tabulation Area (ZCTA) level. The article is concluded in Section 6. Some technical details and plots are given in the Appendix.

## 2. Data

The data analyzed in Sections 4 and 5 are the national Area Deprivation Index (ADI) rankings for the state of South Dakota. The ADI is available as state-level rankings or national-level rankings. The state-level rankings compare all census block groups in the state against one another to provide a ranking of 1–10; the national-level rankings compare all census block groups in a state to all other census block groups in the United State and provide a ranking from 1 to 100 (Kind and Buckingham 2018). In both instances, a higher score represents more socioeconomic disadvantage for that census block unit with 100 being the highest level of disadvantage (1 being the lowest level of disadvantage). It is worth mentioning that the word “ranking” emphasizes relative deprivation compared to other areas, not the size of the gap and is different from statistical ranking. The rankings are available for the years 2015, 2020, and 2021 at the census block group level (Kind and Buckingham 2018; University of Wisconsin School of Medicine and Public Health 2023a, 2023b). The rankings are released to the public to be used in research, program planning, and policy development (Kind and Buckingham 2018). Figure 1 shows spatial maps of the ADI rankings for the three years of available data.

The limited availability of the ADI rankings at both the spatial and temporal levels may present challenges for ADI users. Using the STCOS model, we can produce estimates of the ADI rankings at the desired spatiotemporal support. The goal in this paper is to produce estimates of the ADI rankings at the ZIP Code Tabulation Area (ZCTA) level. Specifically, we use the South Dakota ADI rankings at census block group for 2015, 2020, and 2021 as the source supports and South Dakota ZTCAs for 2021 as the target support.

The South Dakota ZCTAs are used in place of 5-digit ZIP codes. This is because 5-digit ZIP codes are collections of delivery points maintained by the United States Postal Service and do not represent geographic areas. This makes them unsuitable for mapping and many spatial analysis applications. To address this issue, the U.S. Census Bureau constructs ZCTAs, which are geographic estimates for 5-digit ZIP Codes. The most recent ZCTAs are available for the year 2020. More information about ZCTAs can be found in U.S. Census Bureau (2023).

The nature of the ADI rankings presents several challenges. First, the rankings, while close to a normal distribution, are slightly skewed. This violates the normality assumption of the STCOS model. For all years of data, the skewness is −0.30. To address the non-normality of the data, it was transformed using the Box-Cox transformation with a transformation parameter of λ=1.23. Plots of the log-likelihood versus lambda values can be found in Appendix A. The transformed data has a skewness of −0.14. Figure 2 shows the histograms of the original and transformed rankings.

The ADI values are calculated by combining 17 measures across four domains: education, income/employment, housing, and household characteristics from publicly available data, such as the U.S. Census Bureau’s American Community Survey. These measures are standardized and then combined to create a composite score for each neighborhood. Some of these variables do not have the associated variances in the original data. Furthermore, the standardization of the original data would also affect the variance. This makes employing delta method to not be feasible. Therefore, another challenge of the data is that the ADI rankings do not have variances associated with them which are required for constructing the STCOS model. To estimate the variances of the ADI rankings, we employed different techniques to study the effect of variance estimation on the final results. Several of these variance estimation methods required using auxiliary data from the American Community Survey (ACS). The ACS is a survey conducted by the U.S. Census Bureau to collect social, economic, and demographic measures within the United States (U.S. Census Bureau 2020). Specifically, we use median household income, the percentage of the population with no health insurance, and the percentage of the population reporting White race alone. The median household income is a numerical variable in dollars and inflation adjusted and the population with no health insurance and the percentage of the population reporting White race alone are numerical variable between 0 and 100. These variables were chosen because they likely have a significant impact on the ADI rankings. The data was obtained at the census tract level for the years 2015 and 2020 from the Agency for Healthcare Research and Quality Social Determinants of Health database (Agency for Healthcare Research and Quality 2023).

## 3. Statistical Approach

In this section, we review the STCOS model and the methods employed in estimating the variances of the ADI rankings. These methods are K-Means clustering, hierarchical clustering with spatial constraints, multiple linear regression, and finite mixture models.

### 3.1. Spatiotemporal Change of Support Model

Spatiotemporal change of support refers to the process by which data observed or modeled at one spatial and/or temporal resolution are transformed to correspond to a different spatial and/or temporal unit of analysis. In the spatial domain, this involves aggregating or disaggregating measurements across geographic units (e.g., from census block groups to ZCTAs or counties), while in the temporal domain it involves changing the time scale of observation (e.g., from daily to monthly or annual estimates). This change is nontrivial because statistical properties such as the mean, variance, spatial dependence, and uncertainty are not invariant across supports. In public health applications, spatiotemporal change of support is necessary when health outcomes, exposures, and covariates are available at mismatched spatial or temporal resolutions, and failure to account for this mismatch can lead to biased inference, ecological fallacy, or the modifiable areal unit problem (MAUP). Explicitly modeling the change of support allows researchers to harmonize data sources, propagate uncertainty appropriately, and generate valid inferences at the target spatial and temporal scale of interest.

The STCOS model is a method used for making inferences about spatiotemporally correlated data at a spatial and/or temporal support other than the original support. Suppose $Z_{t}^{\left(\ell \right)}(A)$ denote the observed value for the variable of interest at time period ℓ, year t, and spatial region $A\in D_{t}^{\left(\ell \right)}$. The set $D_{t}^{\left(\ell \right)}$ contains the areal units of the source supports with period ℓ and time t. The source supports are the supports where data are available. A fine-level support is also defined. The fine-level support is the smallest resolution for which the data are available. The model translates each of the source supports to the fine-level support during model fitting. After model fitting, estimates at the target support are obtained by translating from the fine-level support.

The observed value, $Z_{t}^{\left(\ell \right)}(A)$, is modeled as the summation of an underlying latent process, $Y_{t}^{\left(\ell \right)}(A)$, and a noise term with a known variance, $V_{t}^{\left(\ell \right)}(A)$. The process $Y_{t}^{\left(\ell \right)}(A)$ is defined as

(1) $$Y_{t}^{\left(\ell \right)}(A)=h(A{)}^{T}\mu_{B}+s_{t}^{\ell }(A{)}^{T}\eta +\xi_{t}^{\ell }(A),$$

where h(A) is the vector of proportions in which A overlaps with each area in the fine-level support. The coefficient $\mu_{B}$ is the change of support coefficient between the fine-level support and the all other supports. The vector $s_{t}^{\ell }(A)$ is a spatiotemporal basis function expansion. This basis function is defined as the aggregation of a continuous-space, discrete-time process over an ℓ-year retrospective period terminating at time t. The coefficient η is a random effects vector with

(2) $$\eta ∼N\left(0,\sigma_{K}^{2}\Sigma \right),$$

where Σ is assumed to be computable from the fine-level support. Lastly, $\xi_{t}^{\ell }(A)$ is a noise term, and we assume $\xi_{t}^{\ell }(A)\overset{\mathrm{iid}}{∼}N(0,\sigma_{\xi }^{2})$. The parameter model is

$$\mu_{B}∼N\left(0,\sigma_{\mu }^{2}I\right),\sigma_{\mu }^{2}∼IG\left(a_{\mu },b_{\mu }\right),$$

$$\sigma_{K}^{2}∼IG\left(a_{K},b_{K}\right),\sigma_{\xi }^{2}∼IG\left(a_{\xi },b_{\xi }\right).$$

A detailed explanation on the parameters, their construction, and their interpretations can be found in Bradley, Wikle, and Holan (2015), Raim et al. (2021), and references therein. The STCOS model can be run in R using the package stcos (Raim et al. 2021).

As previously mentioned, one of the major challenges of the data used in this study is to estimate the values for $V_{t}^{\left(\ell \right)}(A)$. The methodologies used in the estimations of the variances are briefly introduced in the following subsections.

### 3.2. Multiple Linear Regression

Multiple linear regression (MLR) is a traditional approach for understanding the relationship between a response variable and a set of independent variables (Kutner, Nachtsheim, and Neter 2004). The MLR model for n observations and p−1 independent variables can be written in matrix form as

Y=Xβ+ϵ,

where Y is an n×1 vector of the response variable, β is an p×1 vector of regression coefficients, X is an n×p matrix with a column of ones and p−1 columns of the independent variables, and ϵ is an n×1 vector of independent normally distributed error terms with mean 0 and constant variance. The ordinary least squares approach provides unbiased estimator for the regression coefficients. More details about linear regression can be found in Kutner, Nachtsheim, and Neter (2004) and references therein.

### 3.3. Finite Mixture Model

Finite mixture models are a popular statistical technique for modeling data which contains sub-populations (Titterington et al. 1985; Schlattmann 2009; McLachlan, Lee, and Rathnayake 2019). A finite mixture model can be written as a combination of a finite number of different distributions, where each distribution is referred to as a component. Specifically, suppose there are k=1,…,K sub-populations where each one has a probability density function $g_{k}(\cdot )$. The distribution of the response variable, f(y), can be written as a mixture of the distributions of the subpopulations

(3) $$f(y)=\sum_{k=1}^{K}\pi_{k}g_{k}(y),$$

where $\pi_{k}$ are the mixing proportions for each component distribution and must sum to 1. A finite mixture of regression models allows us to perform multiple linear regression while assuming that latent classes exist within the data. Different modeling approaches have been proposed for each component in (3). The FlexMix package in R can be used to create finite mixtures of regression (FMR) models (Gruen and Leisch 2023). For the FlexMix package implementation, each mixture component has its own variance parameter. For more details on finite mixture models see McLachlan, Lee, and Rathnayake (2019) and Grün and Leisch (2007), and references therein.

**Algorithm 1:** K-means Clustering Algorithm

1:	Select K data points at random as seeds. These serve as initial centroids of the K clusters.
2:	Assign each of the data points to the nearest centroid such that it minimizes a criteria. Typically, the criteria is the Euclidean distance.
3:	Recalculate the cluster centroids.
4:	Repeat steps 2 and 3 until the centroids converge and no longer change.

### 3.4. K-Means Clustering

K-means is a well-known algorithm for clustering data (Ahmed, Seraj, and Islam 2020). The process allows us to find latent classes in the data. The aim of K-means clustering is to minimize the following objective function:

(4) $$\sum_{i=1}^{n}\sum_{k=1}^{K}I\left(z_{i}=k\right){\left\|y_{i}-\mu_{k}\right\|}^{2},$$

where n is the number of observed data points, K is the desired number of clusters, $z_{i}$ indicates to which cluster the observed data point $y_{i}$ belongs for i=1,…,n, and $\mu_{k}$ represents the centroid for k=1,…,K. The steps of the K-means approach are detailed in Algorithm 1. Since Algorithm 1 is sensitive to the initial seed choice, the entire process should be repeated with different seeds. For more details on K-means clustering see Hartigan and Wong (1979) and references therein.

### 3.5. Hierarchical Clustering with Spatial Constraints

While K-means clustering can find latent classes within the data, it does not take spatial correlation into account. To address spatial correlation in the clustering process, we employed hierarchical clustering (HC) with spatial constraints (Chavent et al. 2018). This method is based on two dissimilarity matrices, $D_{0}$ and $D_{1}$, and a mixing parameter α∈[0,1]. The matrix $D_{0}$ is computed from the “feature space” of the observations, and $D_{1}$ is computed from the geographic locations of the observations. The parameter α sets the importance of the spatial constraint.

For a given value of α, let $P_{K}^{\alpha }=\left(C_{1}^{\alpha },\ldots ,C_{K}^{\alpha }\right)$ represent the partition of n data points into K clusters. The mixed inertia of cluster $C_{k}^{\alpha }$ for k=1,…,K is defined as

(5) $$I_{\alpha }\left(C_{k}^{\alpha }\right)=(1-\alpha )\sum_{i\in C_{k}^{\alpha }}\sum_{j\in C_{k}^{\alpha }}\frac{1}{2n^{2}\mu_{k}^{\alpha }}d_{0,ij}^{2}+\alpha \sum_{i\in C_{k}^{\alpha }}\sum_{j\in C_{k}^{\alpha }}\frac{1}{2n^{2}\mu_{k}^{\alpha }}d_{1,ij}^{2}$$

where $\mu_{k}^{\alpha }=\sum_{i\in C_{k}^{\alpha }}\frac{1}{n},d_{0,ij}$ is the (i,j)th element of $D_{0}$, and $d_{1,ij}$ is the (i,j)th element of $D_{1}$. The clustering process is detailed in Algorithm 2. This method can be implemented using the package ClustGeo in R (Chavent et al. 2018). We used the Euclidean distance for matrix $D_{1}$ and kept most of the settings to their default setting of the “ClustGeo” package. For more details on the clustering methodology see Chavent et al. (2018) and references therein.

### 3.6. Implementation

All analysis were performed using R Statistical Software (v4.2.0; R Core Team 2021). The K-means clustering and regression were performed using the standard built-in functions in R. The STCOS were performed using “STCOS” package (Raim et al. 2021). The Spatially Constrained Hierarchical Clustering was performed using “ClustGeo” package (Chavent et al. 2018). The finite mixture models were performed using “FlexMix” package (Gruen and Leisch 2023). The Box-Cox transformation was performed using “MASS” package.

## 4. Model Tuning

Several parameters in the STCOS model need to be specified. To find the optimal values of these parameters, the model was tuned using the South Dakota ADI rankings. The parameters of the final model were chosen based on mean square prediction error (MSPE). The MSPE is calculated as

$$\text{MSPE}=\frac{1}{n}\sum_{i=1}^{n}{\left({\overset{ˆ}{Y}}_{i}-Y_{i}\right)}^{2},$$

where n is the number of observations, ${\overset{ˆ}{Y}}_{i}$ is the estimate of the ith observation, and $Y_{i}$ is the observed value of the ith observation.

Four other model evaluation measures were considered when selecting the final model. First was the correlation between the model estimates and observed rankings. For the second measure, a regression line was fit to the estimated and observed rankings. Then the angle of intersection between the regression line and a 45-degree line through the origin was calculated. The third measure was the ratio of performance to deviation (RPD; Kuhn, Vaughan, and Hvitfeldt 2024). The final measure was the maximum distance between the empirical cumulative distribution function (eCDF) of the model estimates and the eCDF of the observed rankings. The best model is desired to minimize the MSPE, simultaneously maximize the correlation and minimize the angle of intersection, have an RPD greater than or equal 2, and minimize the eCDF distance.

**Algorithm 2:** Spatially Constrained Hierarchical Clustering Algorithm

1:	Assign each data point to its own cluster (K=n).
2:	Merge the clusters A and B in $P_{K}^{\alpha }$ such that
	$\delta_{\alpha }\left(A,\;B\right):=I_{\alpha }\left(A\cup B\right)-I_{\alpha }\left(A\right)-I_{\alpha }\left(B\right)$
	is minimized.
3:	Repeat step 2 for K=n−1, n−2,…,1.

The model parameters which needed to be specified were:

1. The estimation method used for the variances of the ADI rankings
2. The hyperparameters needed to calculate $s_{t}^{\ell }(A{)}^{T}$.
3. The structure of the covariance matrix Σ from (2)

For each combination of variance estimation method, hyperparameters, and covariance structure, a model was trained on the 2015 and 2020 ADI rankings and evaluated on the 2021 ADI rankings. Further details on the parameters are outlined in the following subsections.

### 4.1. Variances of ADI Rankings

Since the ADI rankings do not have a direct variances associated with them, the following methods were employed to estimate the variances of the rankings:

- *Yearly:* The sample variances of the ADI rankings for each year t∈{2015,2020,2021}, denoted by $\sigma_{t}^{2}$, were calculated. Then for year t, the rankings were assigned variance estimate $\sigma_{t}^{2}$.
- *KNN:* The variances of the ADI ranking were taken to be the variances of the rankings of each block group and its K-nearest neighbors. The nearest neighbors were determined using the Euclidean distances between the central points of the block groups. We considered values of K=3,4,5. In cases where the variance was zero, the variance was replaced with the K+1 nearest neighbor variance.
- K-*Means:* The block groups were clustered based on the ACS variables using Algorithm 1. The variance of the ADI rankings within each cluster was assigned to the rankings within the cluster. For K>23, the variance in at least one cluster was equal to zero, so we considered K=13,18,23 for the number of clusters.
- *HC:* The block groups were clustered based on the ACS variables using Algorithm 2. The variance of the ADI rankings within each cluster was assigned to the rankings within the cluster. For K>22, the variance in at least one cluster was equal to zero, so we considered K=12,17,22 for the number of clusters. For the mixing parameter we considered α=0.1,0.2,0.3.
- *MLR:* The ADI rankings were predicted based on the ACS variables using a multiple linear regression model for each year of data. The variances of the rankings were taken to be the absolute value of the residuals.
- *FMR:* The ADI rankings were predicted based on the ACS variables using a finite mixture regression model with two components for each year of data. The number of components was chosen based on the AIC and BIC of the FMR models. The variances of the rankings were taken to be the absolute value of the residuals after assigning the observations to one of the subgroups using maximum posterior probabilities.

### 4.2. Specification of Basis Functions

Computation of the vector $s_{t}^{\ell }(A{)}^{T}$ in (1) requires specifying spatial knots, temporal knots, a spatial radius $w_{s}$, and a temporal radius $w_{t}$. The number of knot points considered were $r_{s}=25,\;50,\;100,\;150,\;200,\;250,\;300$, and 350. The spatial knots were placed using space filling design. The spatial radius $w_{s}$ was chosen by using quantiles of the distance between all spatial knots points. We considered the quantiles of q=0.05,0.10,0.15,0.20,0.25, and 0.30. Since the data are not available every year, using an evenly-spaced grid of temporal knots would lead to place the knots on years that we did not have any data. Therefore, the temporal knots were placed at the years 2015, 2020, and 2021. The temporal radius $w_{t}$ was chosen to be one.

### 4.3. Specification of Random Effect Covariance

There are several possible structures for the covariance matrix, Σ, from (2) based on the assumptions of the random effects vector η. Three methods were used to compute Σ. These methods were based on the following assumptions:

- **I:** No spatial or temporal correlation
- **S:** Correlation across space but independence across time
- **RW:** Correlation across space and a nonstationary random walk process across time

The optimal structure for Σ was identified through a comparison of three candidate specifications. For brevity, only the corresponding results for the best one is reported. For further details of the computation of Σ see Bradley, Wikle, and Holan (2015) and Raim et al. (2021).

## 5. Results and Discussion

In this section, we discuss the results of the model tuning and use the final model to estimate the ADI rankings for South Dakota ZCTAs in the year 2021. All analysis were run on South Dakota State Roaring Thunder High-Performance Computing (HPC) with 40 CPU Intel Xeon Gold 6342 @ 2.80GHz and 100 GB of RAM. The results for each case are based on 25,000 posterior samples with the first 5000 as a burn-in period and thinning parameter of 10. Table 1 summarizes the results of the best model for each of the variance estimation methods for both the original and transformed ADI rankings. For all variance estimation methods, the optimal computation of Σ resulted from assuming spatial covariance. The basis function parameters were chosen based on the interior point with the lowest MSPE. The interior points were considered to be the elements of the Cartesian product of $r_{s}$ and q which did not include a minimum or maximum value for either parameter. This was done because the MSPE values by basis function parameters began to plateau as they decreased. The optimal values of $r_{s}$ and q were found to be 300 and 0.25, respectively for all models. See Appendix B for plots of the MSPE values by basis function parameters for each variance estimation method.

The MLR variance method produced the best model estimates for both the original and transformed data with the FMR variance method a close second. The models for the original and transformed data performed similarly, showing the model is not overly sensitive to some skewness in the data. Interestingly, for the HC variance method, as the number of clusters used increased, the models performed better, but for the K-Means method, as the number of clusters decreased, the models performed better.

The models listed for the original data in Table 1 were used to fit final models. All data from 2015, 2020, and 2021 was used during the final model fittings. Estimates were then produced at the ZCTA level in the state of South Dakota for the year 2021. Figure 3 shows the spatial plots of the resulting estimates and the associated posterior standard deviations for the MLR model. The ZTCAs with the large standard deviations are in an area missing data at the block group level for all three years. Plots of the other model estimates and their associated posterior standard deviations can be found in Appendix C. Figure 4 shows the distributions of the 2021 ADI rankings and the MLR model estimates. The standard deviation of the estimates was less than that of the ADI rankings, but the estimates were able to capture the center and skewness of the rankings.

## 6. Conclusions

Social determinants of health are recognized as important factors of overall health and wellness. Understanding factors related to economic stability, education access and quality, healthcare access and quality, housing security and environment, and social and community context is valuable to understanding how long and how well we live. There are public datasets available providing discrete variables for SDOH. One of the variables we focused on is the ADI rankings because it is a multidimensional index that includes socioeconomic variables gathered for the U.S. Census. The ADI rankings are correlated over space and time. A challenge of analyzing spatiotemporal data are that the support of the observed data does not always match the desired support. Finding a method to produce estimates for spatiotemporal SDOH variables will provide more accurate insights into the impact of socioeconomic factors on overall health.

The STCOS method can be used to produce estimates for spatiotemporal variables of interest at a desired support and provide measures of uncertainty. This study focused on addressing challenges associated with using the STCOS model on Area Deprivation Index (ADI) rankings in South Dakota. The STCOS methodology requires that the observed data values have known variances. For ADI rankings, these variances are not readily available. In addition, the methodology assumes reasonably Gaussian data; however, the ADI rankings are skewed. We studied the effect of several variance estimation methods for the observed values, and the effect of transforming the data to achieve a more normal distribution. Using the proposed variance estimation methods on the original and transformed data, we implemented the STCOS model and performed model tuning. It was found that the skewness of the data had little effect on the results of the model. The final model with the optimal variance estimation method was used to estimate ADI rankings at the ZCTA level in South Dakota for the year 2021.

Although it is difficult to identify South Dakota–specific programs, policy changes, or interventions that explicitly cite the ADI as a foundational tool, South Dakota is involved in many national and local initiatives that align with its principles (United Health Foundation 2024). One such national initiative is the Centers for Medicare and Medicaid Services (CMS) Accountable Care Organization Realizing Equity, Access, and Community Health (REACH) Model, which emphasizes health equity for underserved Medicare populations by encouraging care coordination, improving quality, and ultimately reducing costs while improving patient outcomes (Centers for Medicare and Medicaid Services 2022). While the ADI is not consistently identified as a standalone policy driver in South Dakota, its underlying concept, using measures of neighborhood disadvantage to guide resource allocation, is embedded in how the South Dakota Department of Health defines rurality and evaluates health needs for programs focused on improving care in underserved communities (Powell, Sheehy, and Kind 2023; South Dakota Department of Health (n.d.)).

The methods proposed in this article have their own limitations that provide opportunity for improvement and expansion. The selection process of the ACS variables used in several of the variance estimation methods could be refined using variable screening techniques. A different set of independent variables may result in better model estimates. Furthermore, the variance estimates used in this study are plug-in estimates because ADI rankings are not typically released with known sampling variances. Although the proposed approaches provide practical ways to incorporate uncertainty into the STCOS framework, they remain approximations and may influence the resulting ZCTA-level estimates. Future work should examine the sensitivity of policy-relevant rankings to alternative variance specifications and develop methods that more fully propagate uncertainty from the variance estimation stage. In addition, the South Dakota analysis should be interpreted as a demonstration of the change-of-support framework rather than as an evaluation of a specific public policy intervention. The estimated ZCTA-level ADI rankings may help identify underserved communities and inform equitable resource allocation, but this study does not directly assess downstream policy outcomes. Future research could apply the proposed framework to specific health equity initiatives, program eligibility rules, or allocation decisions to better quantify its practical policy impact. Finally, it is possible to modify the STCOS model to allow for distributions of data other than Gaussian. Work on a STCOS model with a skewed normal assumption is currently underway.

## Supplementary Material

Supp 1

The Supplementary Materials provide a description of ZIP Codes versus ZIP Code Tabulation Areas (ZCTAs), the results of the Box-Cox transformation, model-tuning plots showing MSPE by basis-function parameters for each variance-estimation method using both original and transformed ADI rankings, and a census-tract-level plot comparing observed and predicted ADI values for 2021.

Supplementary materials for this article are available. Please go to www.tandfonline.com/uspp.

## Figures and Tables

**Figure 1.**
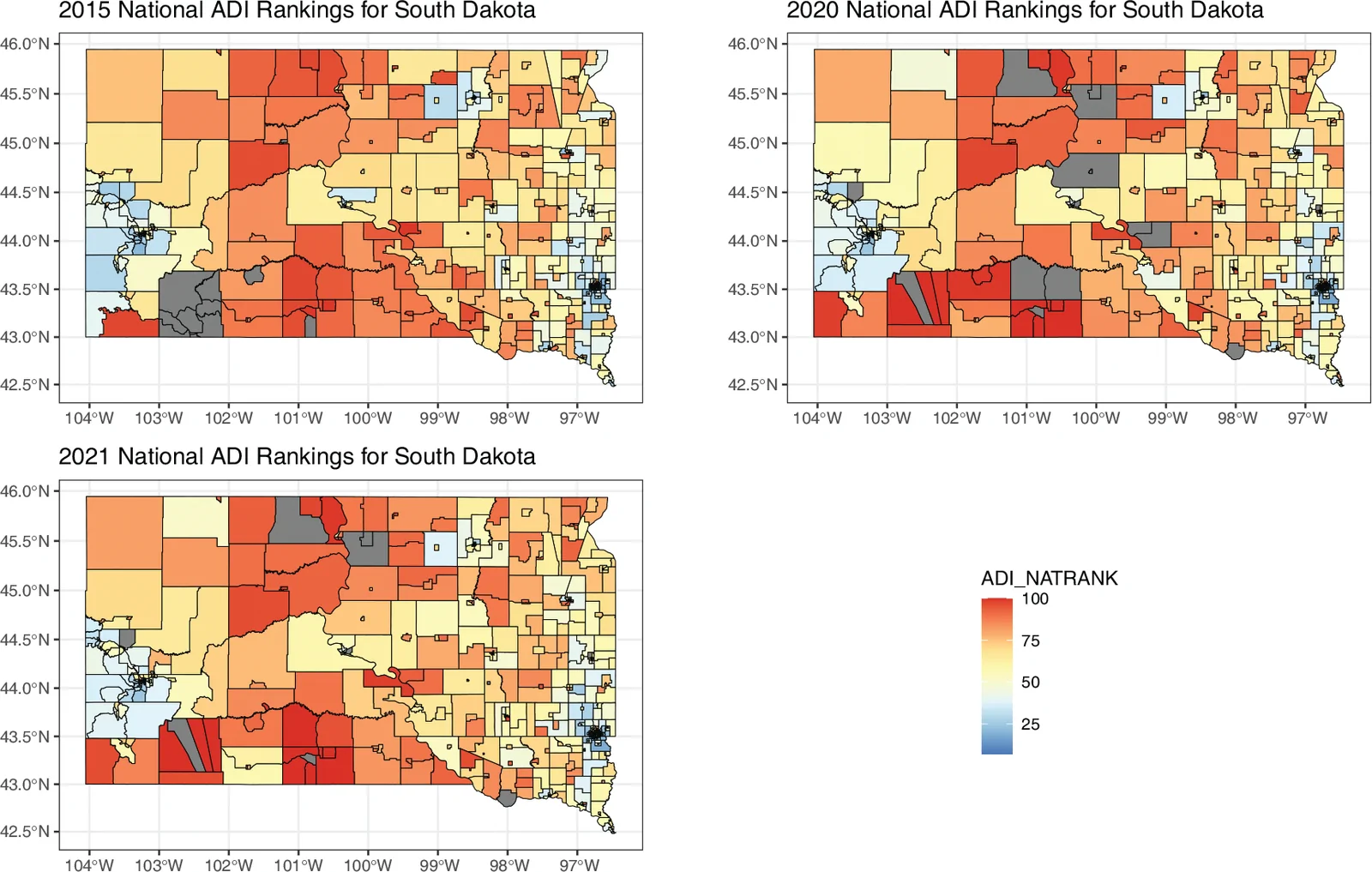
National ADI rankings in South Dakota for the years 2015, 2020, and 2021. ADI rankings were not publicly available for the areas with gray shading.

**Figure 2.**
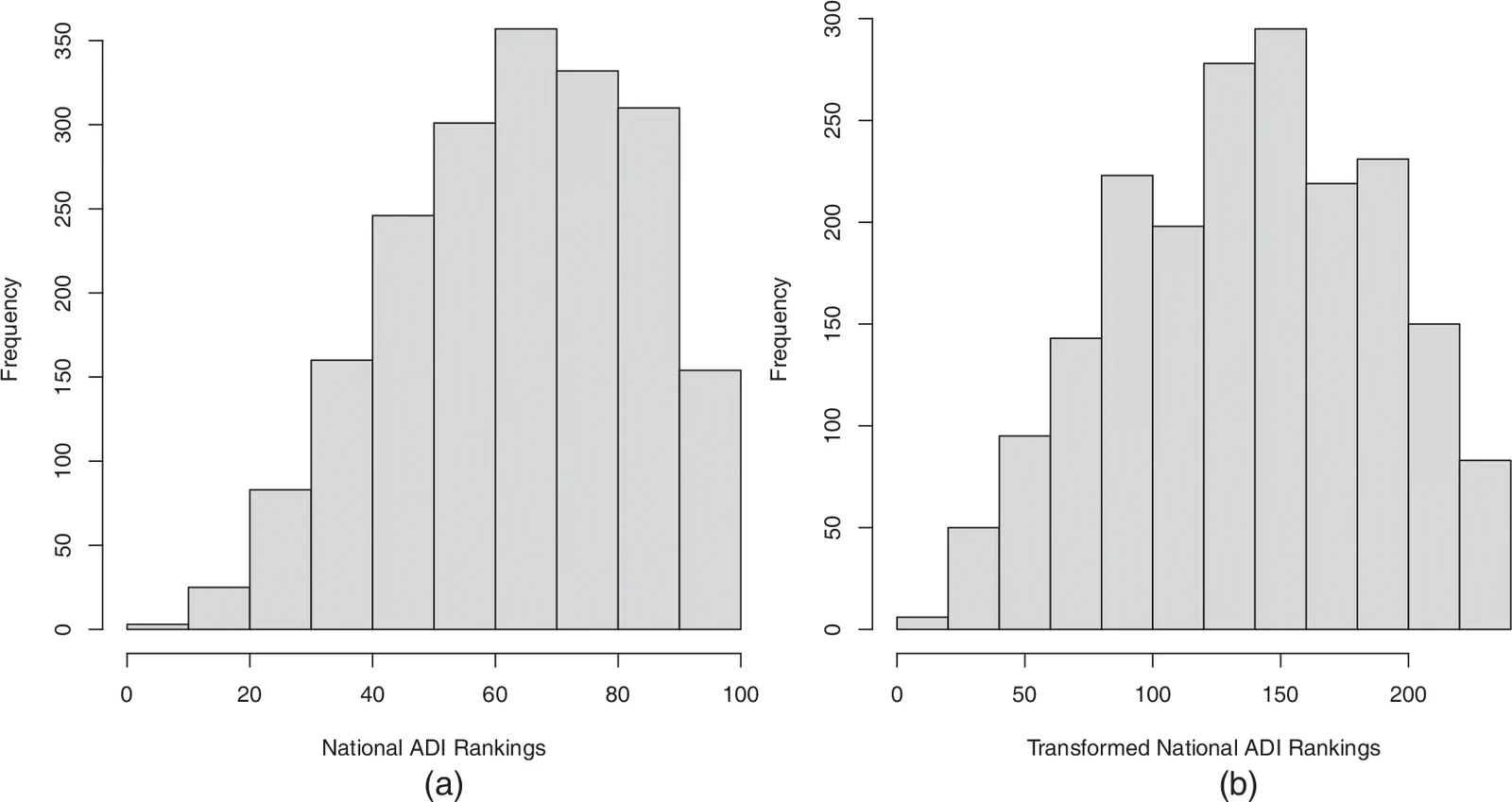
(a) Histogram of original ADI rankings for all years. (b) Histogram of transformed ADI rankings for all years.

**Figure 3.**
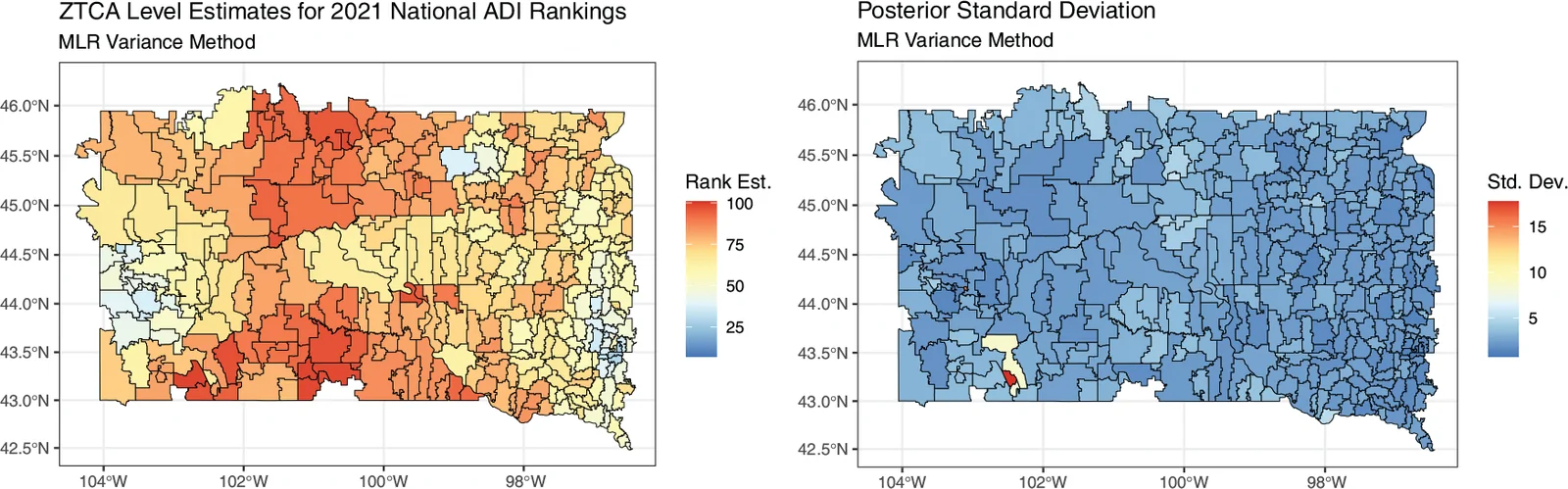
MLR model estimates for 2021 South Dakota ZCTA ADI rankings and the associated posterior standard deviations.

**Figure 4.**
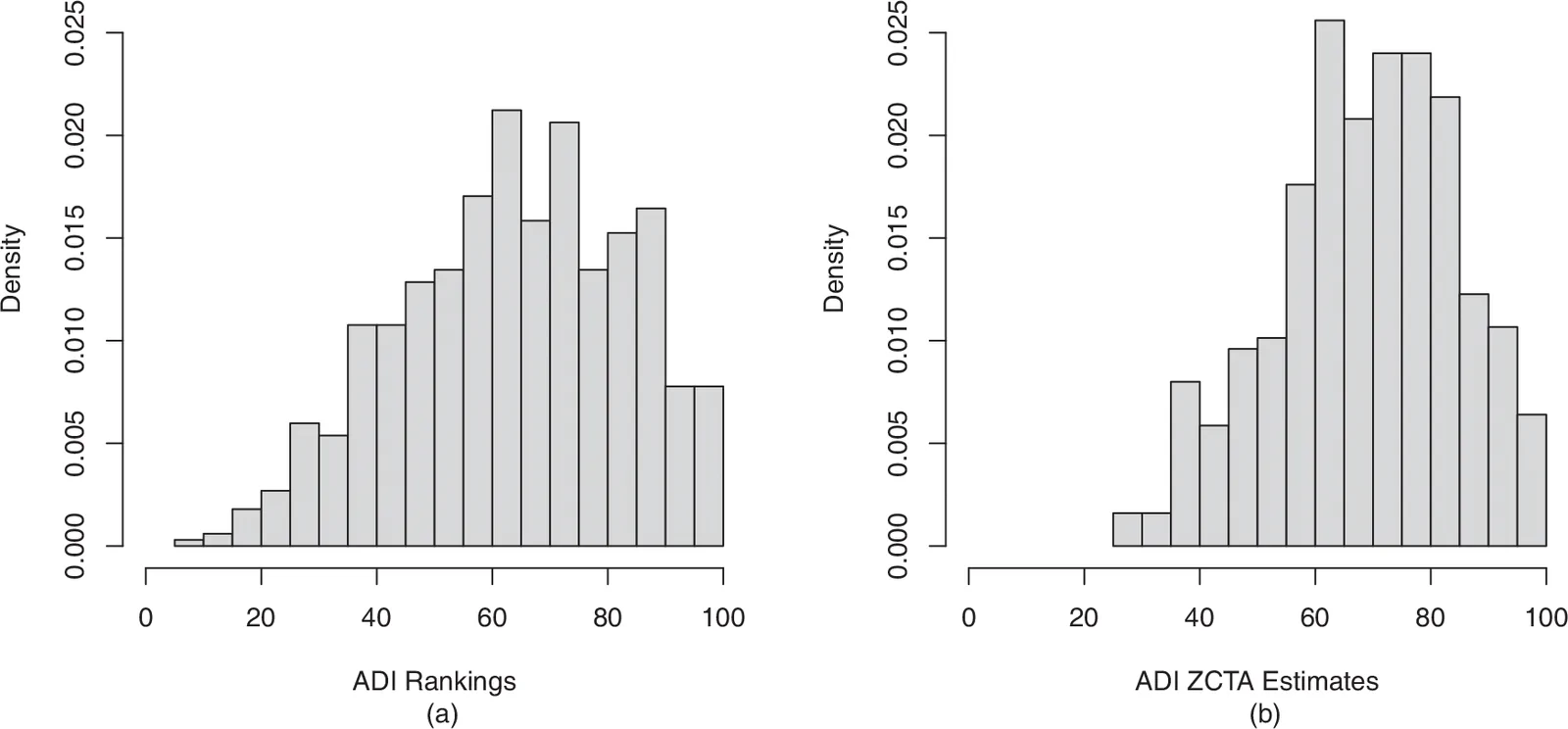
(a) Histogram of 2021 South Dakota block group ADI rankings. (b) Histogram of 2021 ZCTA MLR model estimates.

**Table 1.** Comparison of top models for each variance estimate method based on MSPE, the correlation between the estimated and observed values (*ρ*), the angle of intersection between the regression line fit to the estimated and observed values and a 45-degree line (ang.), the RPD, the eCDF distance, and computing time in minutes for 25,000 posterior sample draw.

Data	Variance	MSPE	*ρ*	Ang.	RPD	eCDF	Time

Original	Yearly	149.62	0.939	23.70	1.60	0.25	6.07
	KNN (K = 3)	60.38	0.924	8.11	2.51	0.10	6.19
	***K***-Means (K = 13)	53.71	0.944	9.15	2.67	0.13	6.19
	HC (K = 22, *α*=0.2)	50.36	0.940	7.65	2.75	0.11	5.63
	MLR	40.81	0.945	4.18	3.06	0.07	6.66
	FMR	42.62	0.944	4.48	2.99	0.07	6.68

Transformed	Yearly	154.19	0.937	24.17	1.57	0.25	7.06
	KNN (K = 3)	60.14	0.925	8.12	2.52	0.10	7.28
	***K***-Means (K = 13)	55.00	0.943	9.37	2.63	0.13	6.83
	HC (K = 22, *α*=0.2)	51.14	0.940	7.82	2.73	0.11	7.44
	MLR	41.59	0.945	4.50	3.03	0.08	6.58
	FMR	41.70	0.945	4.54	3.02	0.07	6.81

NOTE: All models used 300 spatial knots, a spatial radius quantile of 0.25, and the spatial prior for the covariance structure.
