# Supplementary material for: Estimation of Area Deprivation Index Rankings in South Dakota Using Spatiotemporal Change of Support Method
Source: Stat Public Policy (Phila). Author manuscript; Available in PMC 2026 Oct 15. (PMC13664641; doi:10.1080/2330443x.2026.2704953)
Supplement: Supplementary file 1 [file NIHMS2211749-supplement-Supp_1.pdf]

Supplementary Materials: Estimation of Area Deprivation Index Rankings in South Dakota Using Spatiotemporal Change of Support Method

1 Appendix A: ZIP Codes vs. ZIP Code Tabulation Area (ZCTA)

ZIP codes are created and maintained by the United States Postal Service for the purpose of mail delivery and represent delivery routes rather than fixed geographic areas, which means they may correspond to a single building, a set of streets, or post office boxes and can change frequently based on operational needs. In contrast, ZIP Code Tabulation Areas (ZCTAs) are geographic units developed by the U.S. Census Bureau to approximate ZIP Codes for statistical and mapping purposes; ZCTAs are constructed by aggregating census blocks that share the same predominant ZIP Code, making them stable, area-based representations suitable for demographic analysis, public health research, and spatial data visualization, even though they do not perfectly match USPS ZIP Code boundaries (U.S. Census Bureau, 2023).

ZCTA-level estimates are frequently used in public health practice and research because they offer a practical balance between geographic resolution, data availability, and statistical reliability. Although census block groups provide finer spatial detail, many health outcomes and indicators are not released at the block-group level due to confidentiality concerns, small population sizes, and data suppression policies, particularly for rare events or sensitive conditions (Krieger et al., 2002). As a result, ZCTAs often represent the most granular geography at which stable, population-based health data are publicly available. Moreover, ZCTAs align closely with how health data are collected and disseminated, as many administrative, clinical, and surveillance datasets are indexed by ZIP Code rather than census geography. Examples of health data commonly available only at the ZIP Code or ZCTA level include CDC PLACES estimates of chronic disease prevalence and health behaviors (Centers for Disease Control and Prevention, 2023), hospital discharge and emergency department utilization data (Agency for Healthcare Research and Quality, 2023), mortality and vital statistics (National Center for Health Statistics, 2023), infectious disease surveillance such as COVID-19 case and vaccination rates (Centers for Disease Control and Prevention, 2022), and insurance claims data from Medicare, Medicaid, and commercial payers (Centers for Medicare & Medicaid Services, 2023). Consequently, the use of ZCTA-level estimates facilitates data linkage

across sources and supports actionable, policy-relevant analyses, even though these units are coarser than census block groups.

2 Appendix B: Box-Cox Transformations

This section provides the results from Box-Cox transformation for ADI rankings for all years combined in Figure 1 and individual years in Figures 2, 3, and 4.

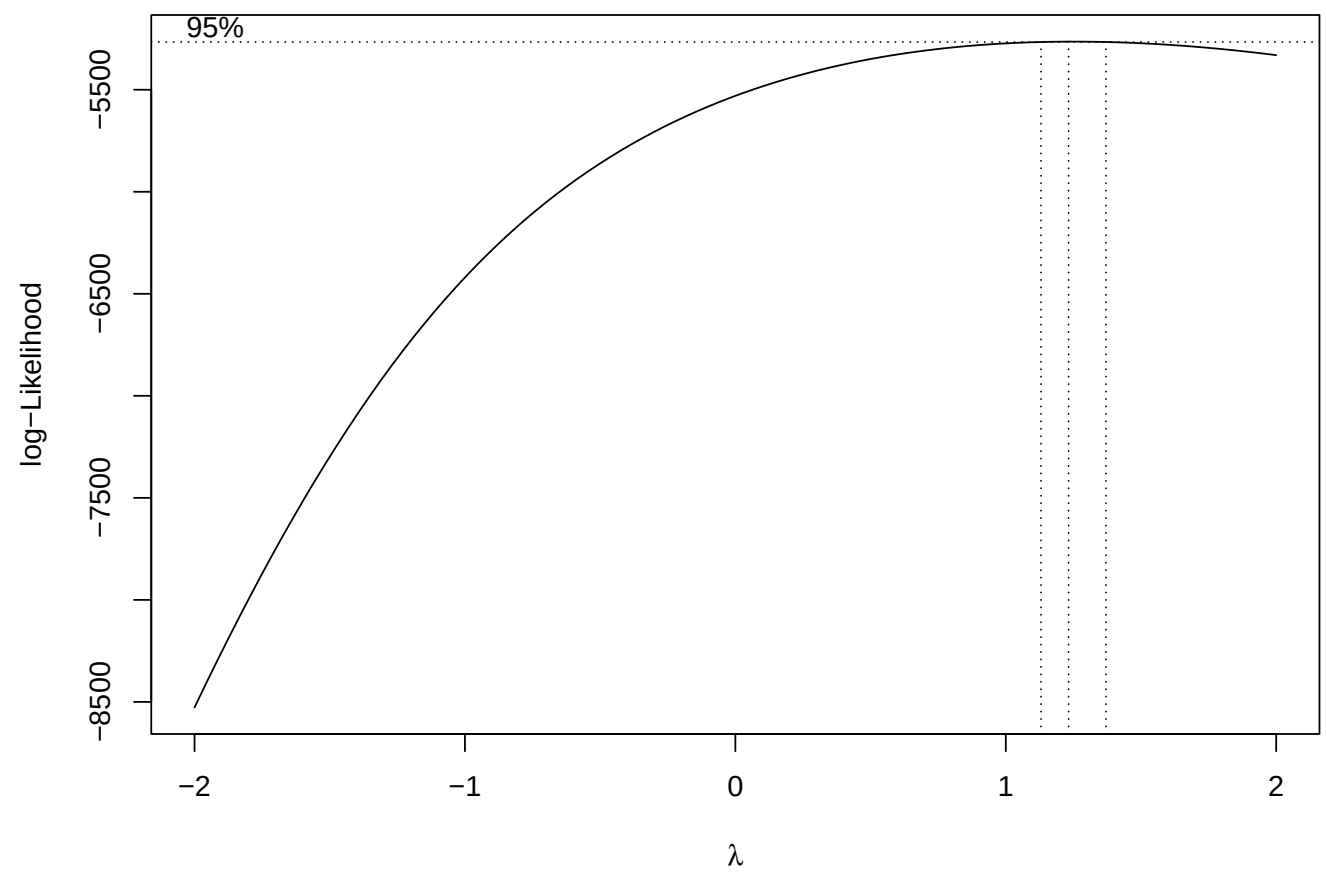

Figure 1: Box Cox transformation of ADI rankings for all years: log-likelihood vs. lambda with a 95% confidence interval. Optimal value of $\lambda = 1.23$.

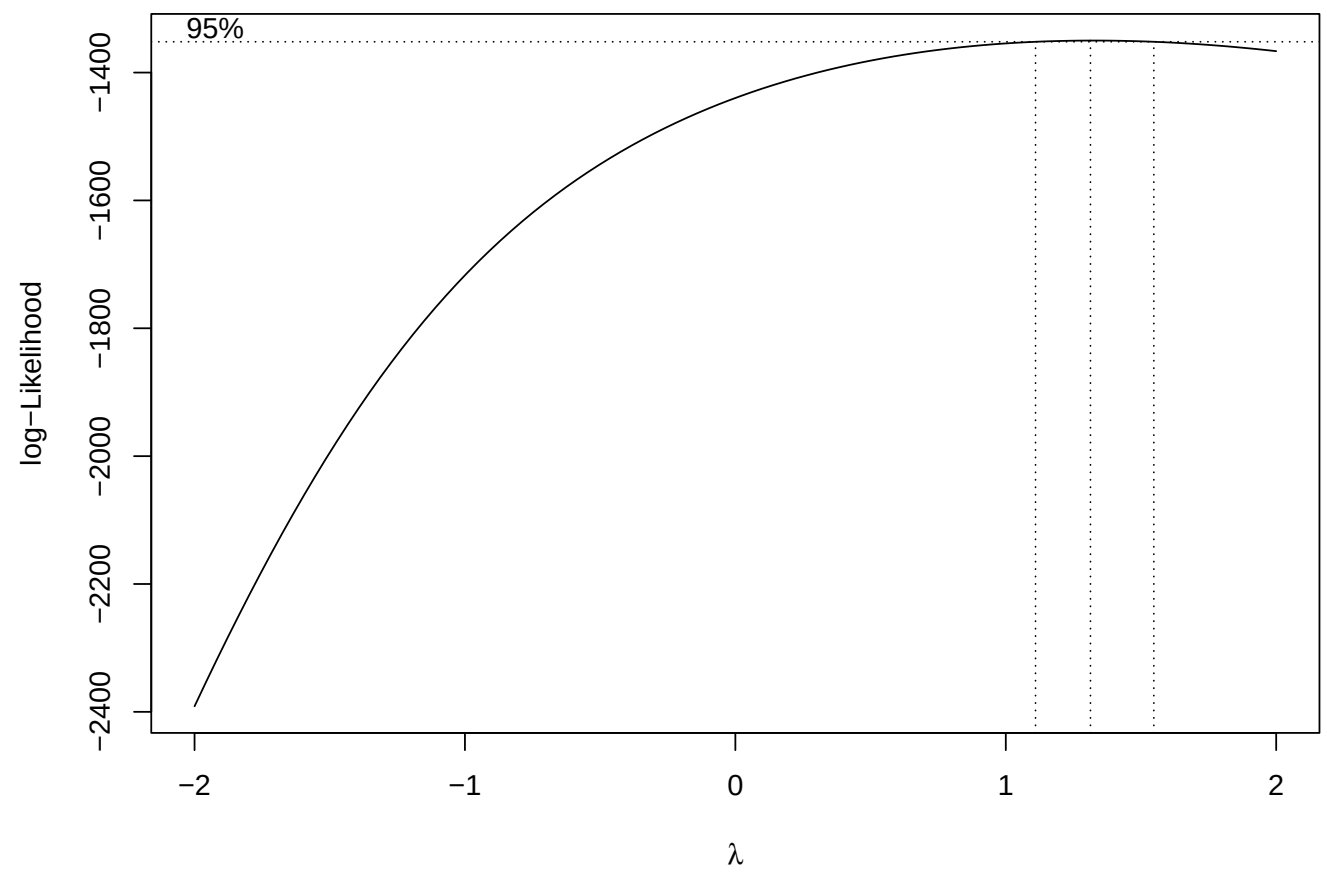

Figure 2: Box Cox transformation of ADI rankings for year 2015: log-likelihood vs. lambda with a 95% confidence interval. Optimal value of $\lambda = 1.31$.

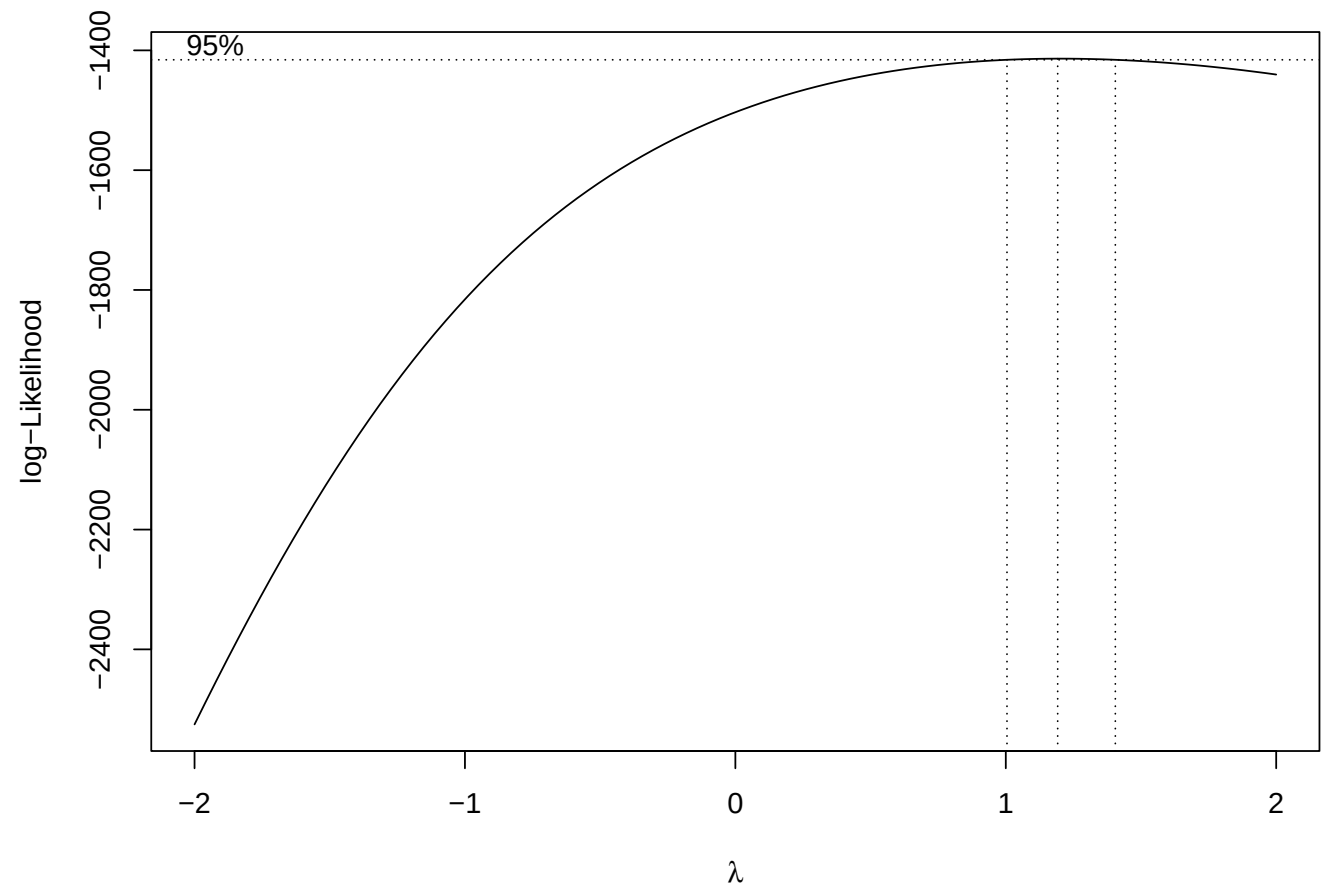

Figure 3: Box Cox transformation of ADI rankings for year 2020: log-likelihood vs. lambda with a 95% confidence interval. Optimal value of $\lambda = 1.19$.

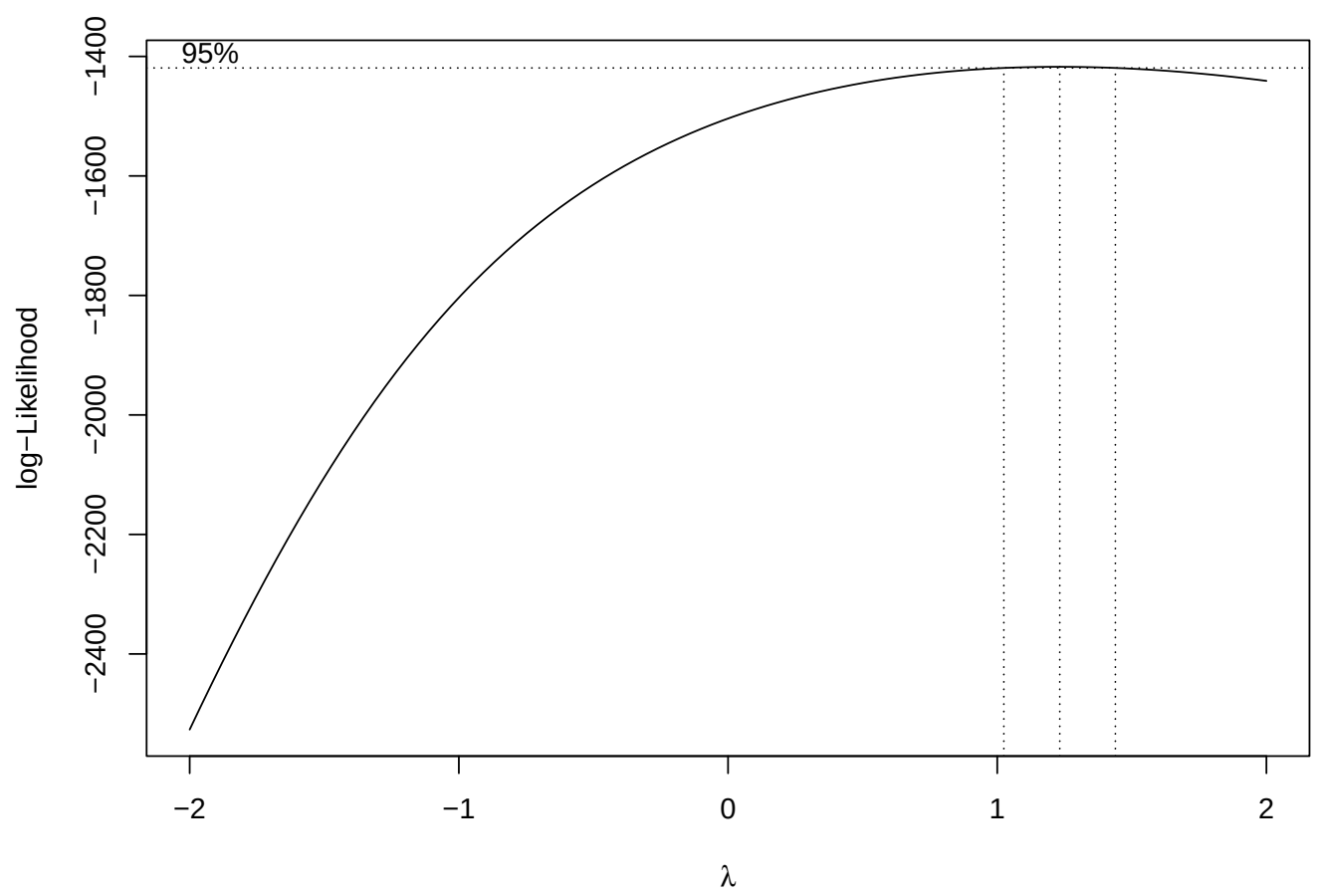

Figure 4: Box Cox transformation of ADI rankings for year 2021: log-likelihood vs. lambda with a 95% confidence interval. Optimal value of $\lambda = 1.23$.

3 Appendix C: MSPE Plots

This section provides plots of results from the model tuning. Figures 5 - 10 show MSPE by basis function parameters for each variance estimation method. Figures 12 - 16 show MSPE by basis function parameters for each variance estimation method using the transformed ADI rankings.

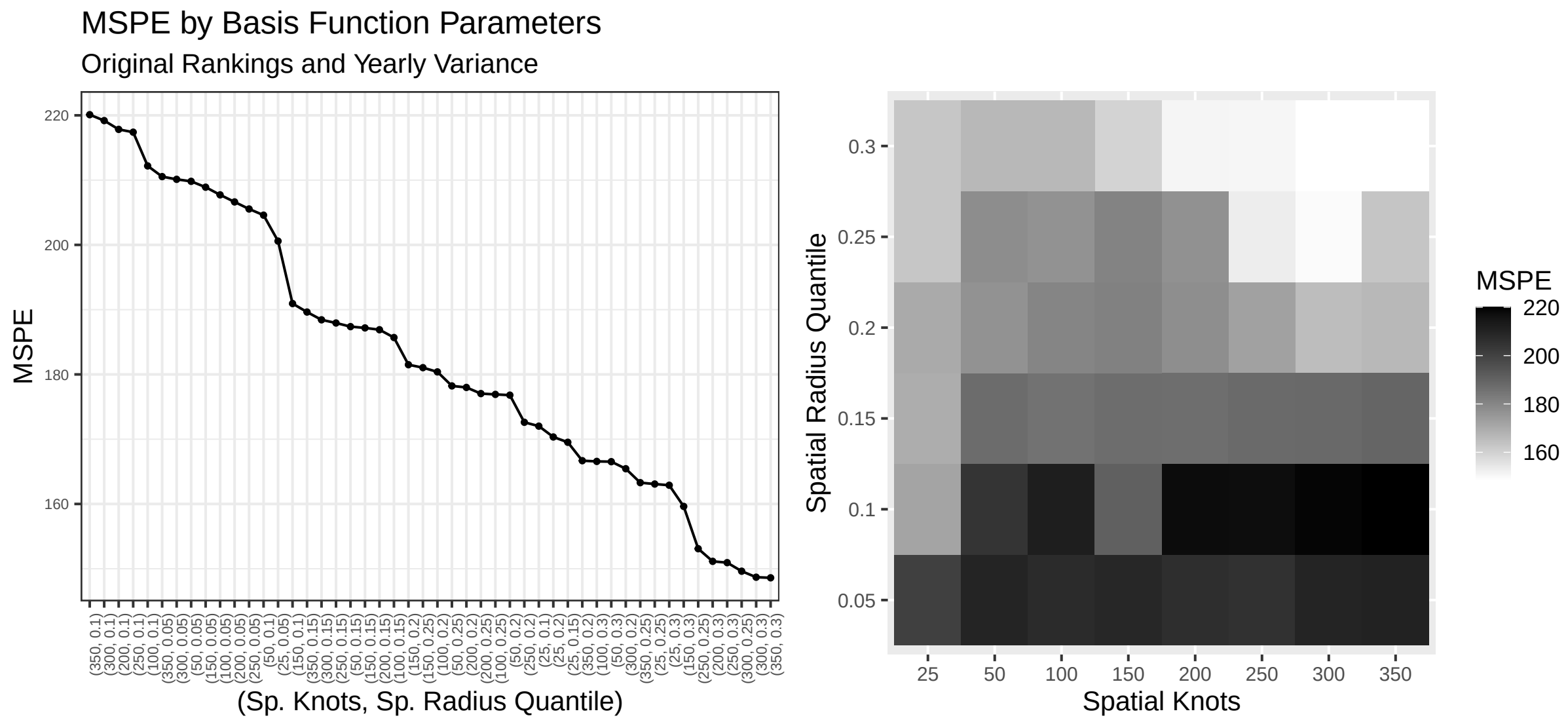

Figure 5: MSPEs by basis function parameters for models using original ADI rankings, the yearly variance estimate method, and the S method for the computation of K.

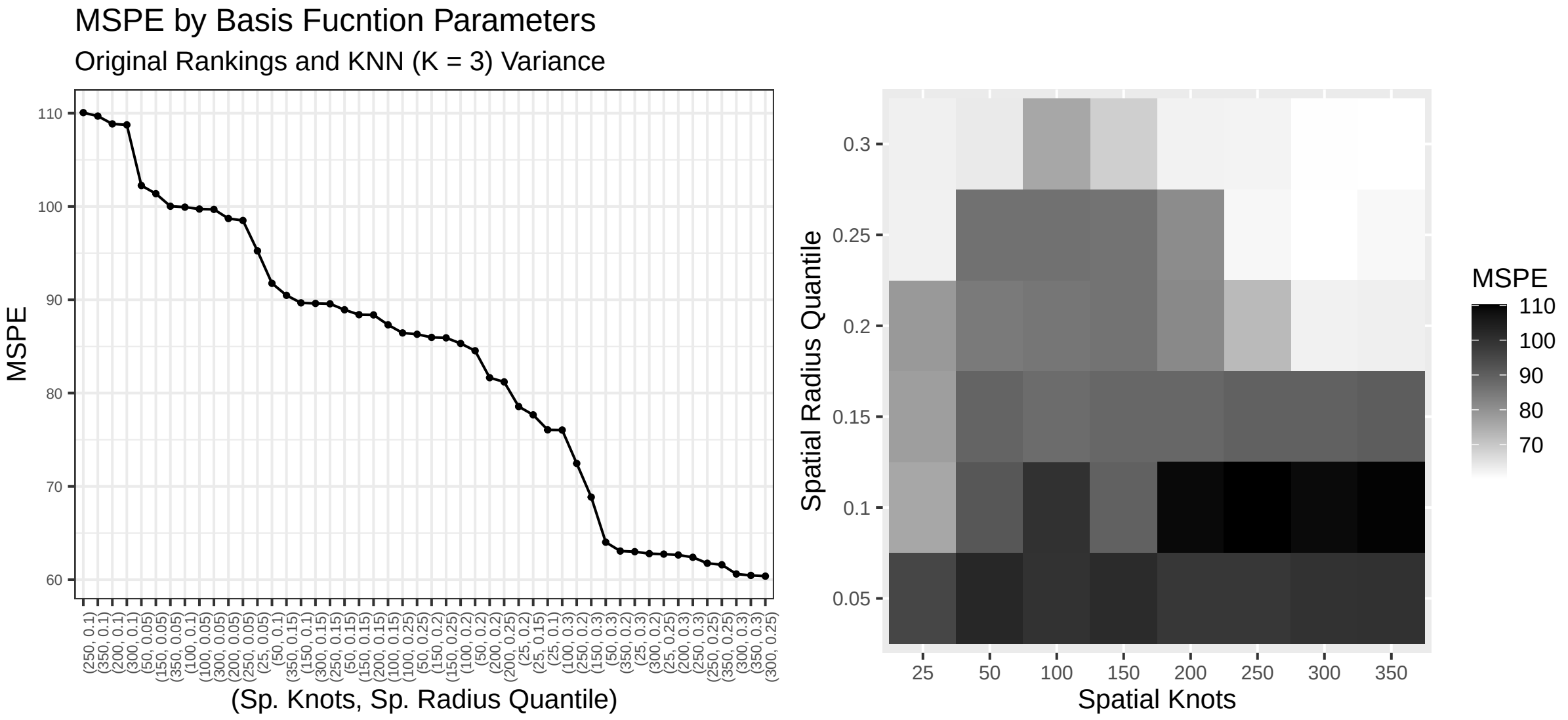

Figure 6: MSPEs by basis function parameters for models using original ADI rankings, the KNN (K = 3) variance estimate method, and the S method for the computation of K.

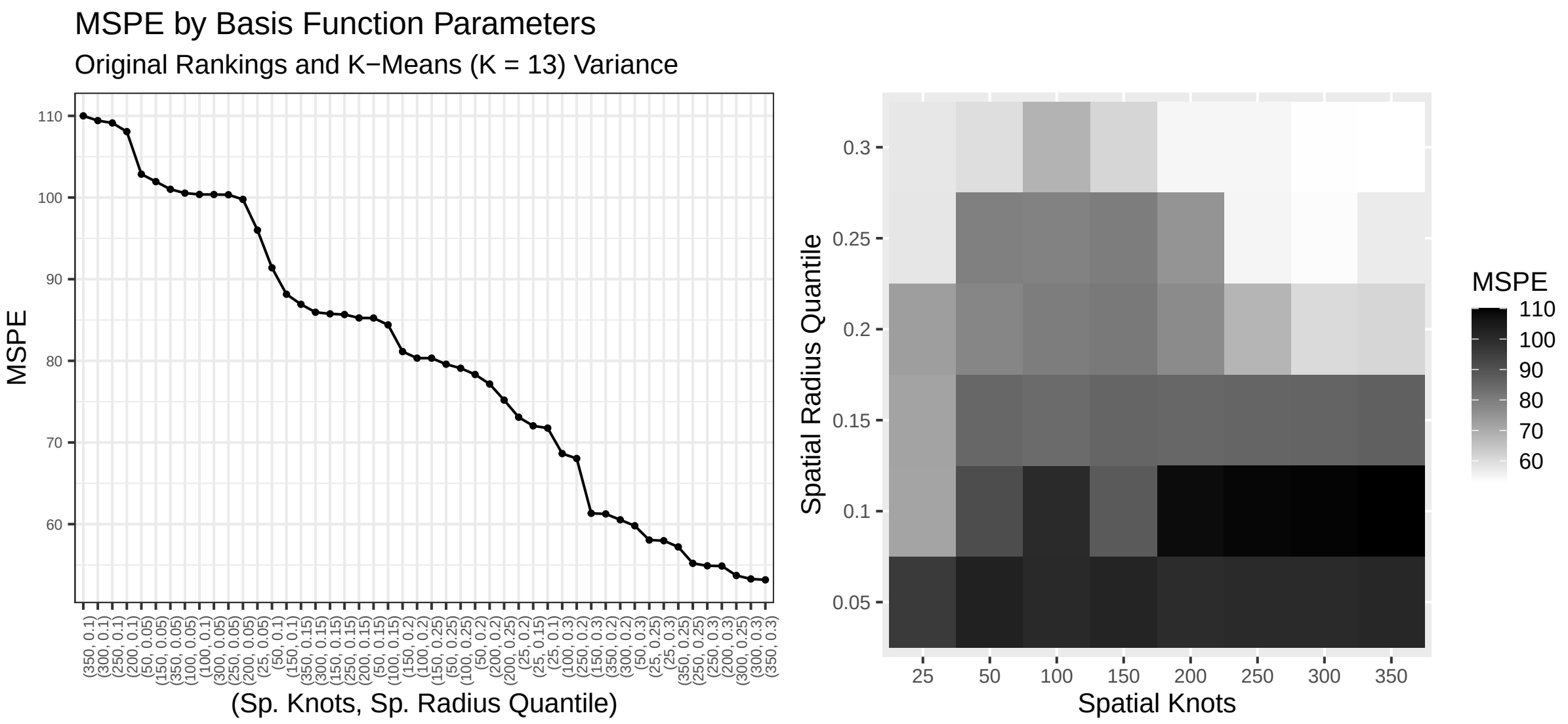

Figure 7: MSPEs by basis function parameters for models using original ADI rankings, the K-Means (K = 13) variance estimate method, and the S method for the computation of K.

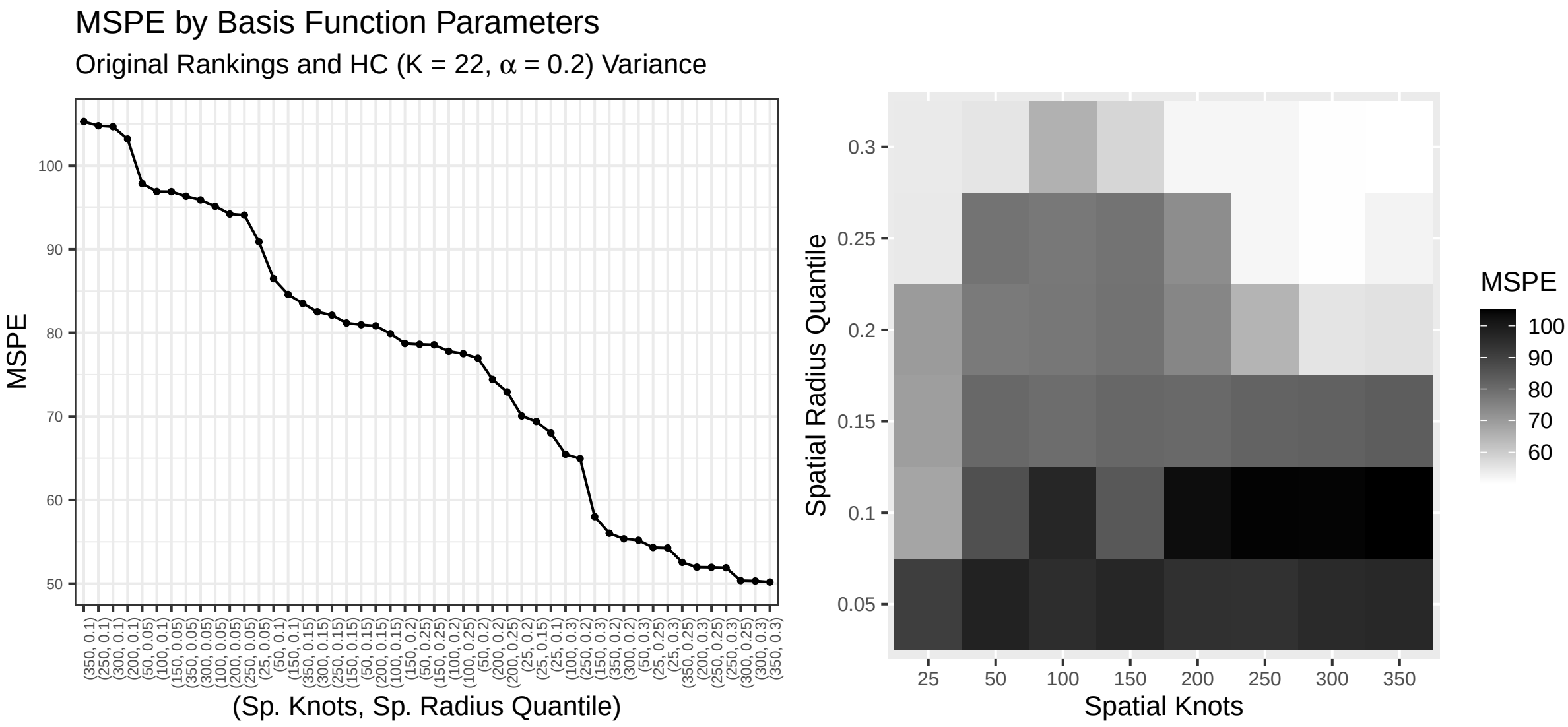

Figure 8: MSPEs by basis function parameters for models using original ADI rankings, the HC (K $= 22$, $\alpha = 0.2$) variance estimate method, and the S method for the computation of K.

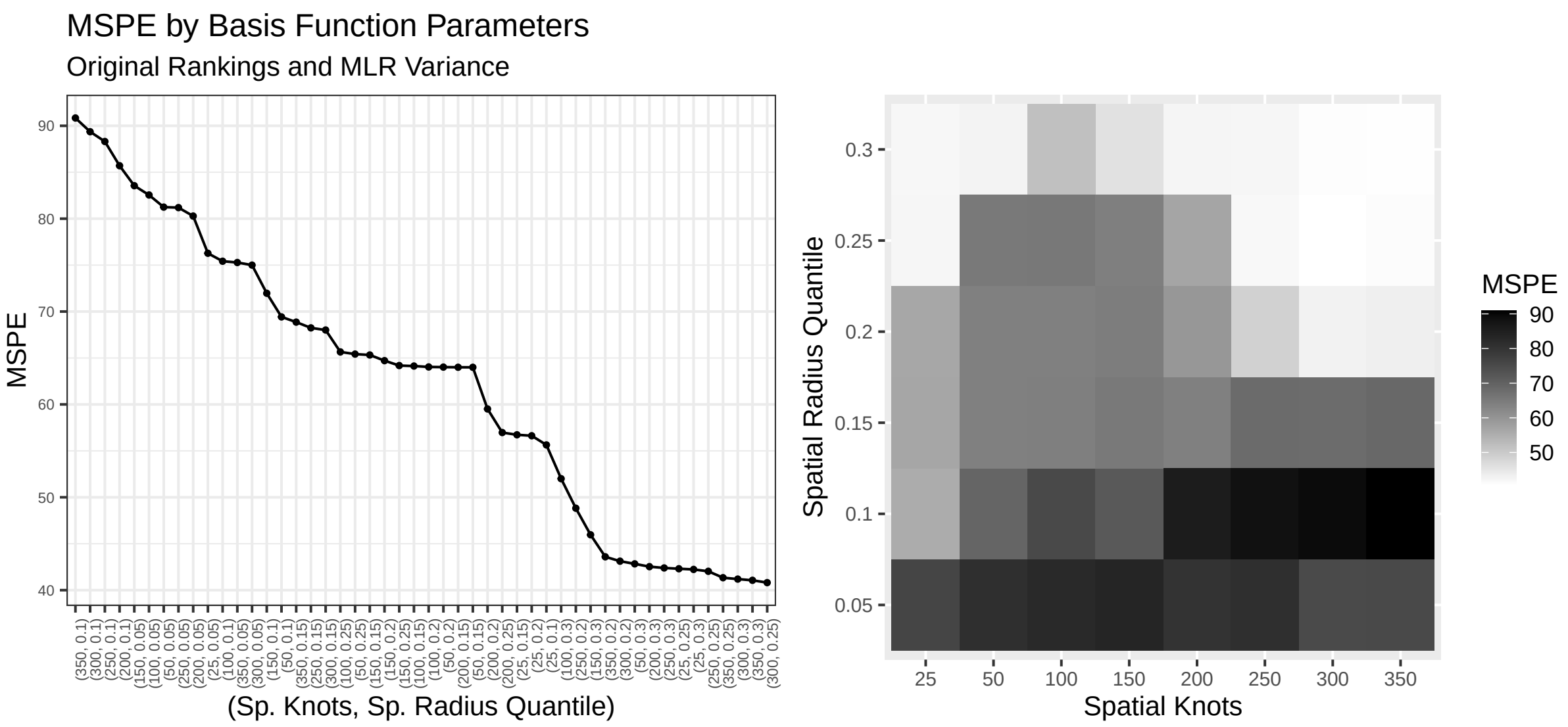

Figure 9: MSPEs by basis function parameters for models using original ADI rankings, the MLR variance estimate method, and the S method for the computation of K.

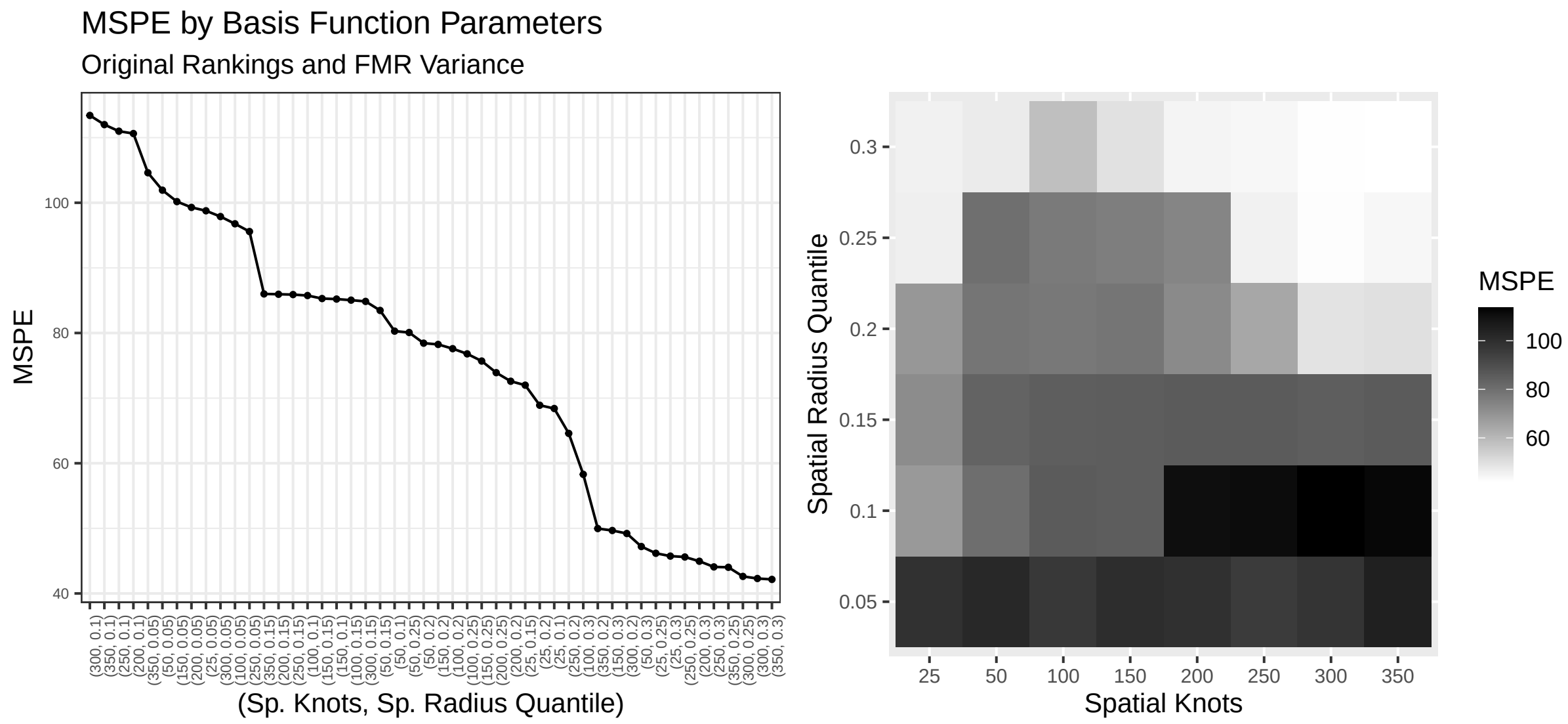

Figure 10: MSPEs by basis function parameters for models using original ADI rankings, the FMR variance estimate method, and the S method for the computation of K.

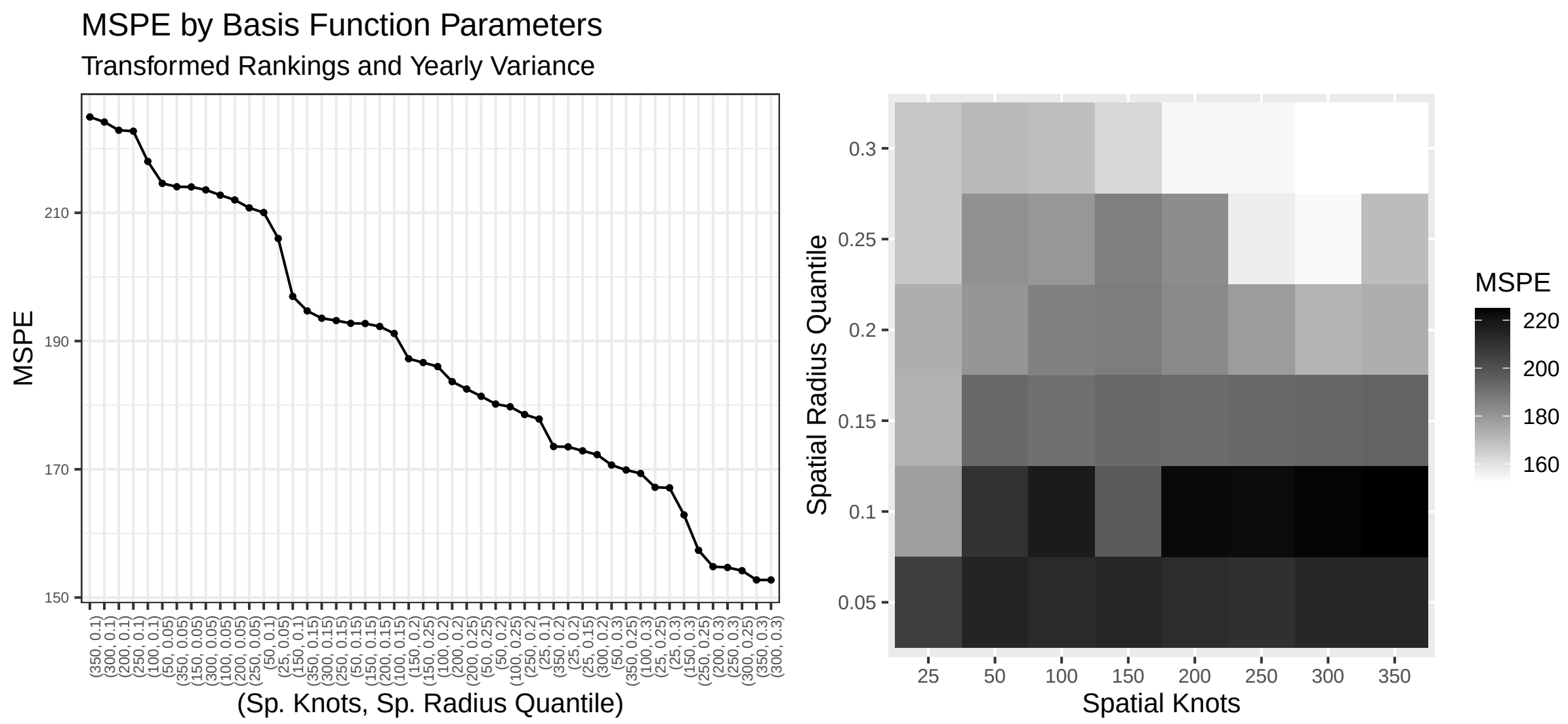

Figure 11: MSPEs by basis function parameters for models using transformed ADI rankings, the yearly variance estimate method, and the S method for the computation of K.

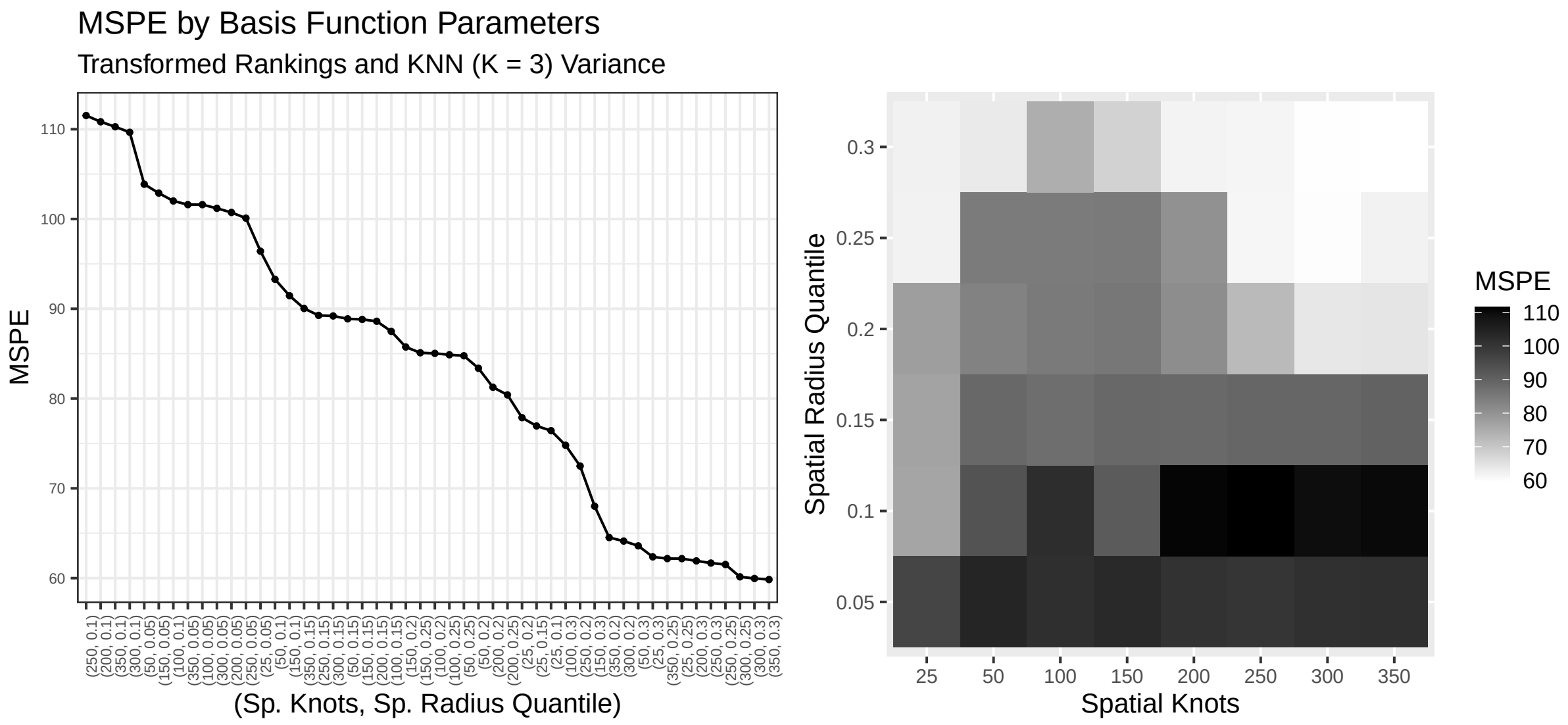

Figure 12: MSPEs by basis function parameters for models using transformed ADI rankings, the KNN (K = 3) variance estimate method, and the S method for the computation of K.

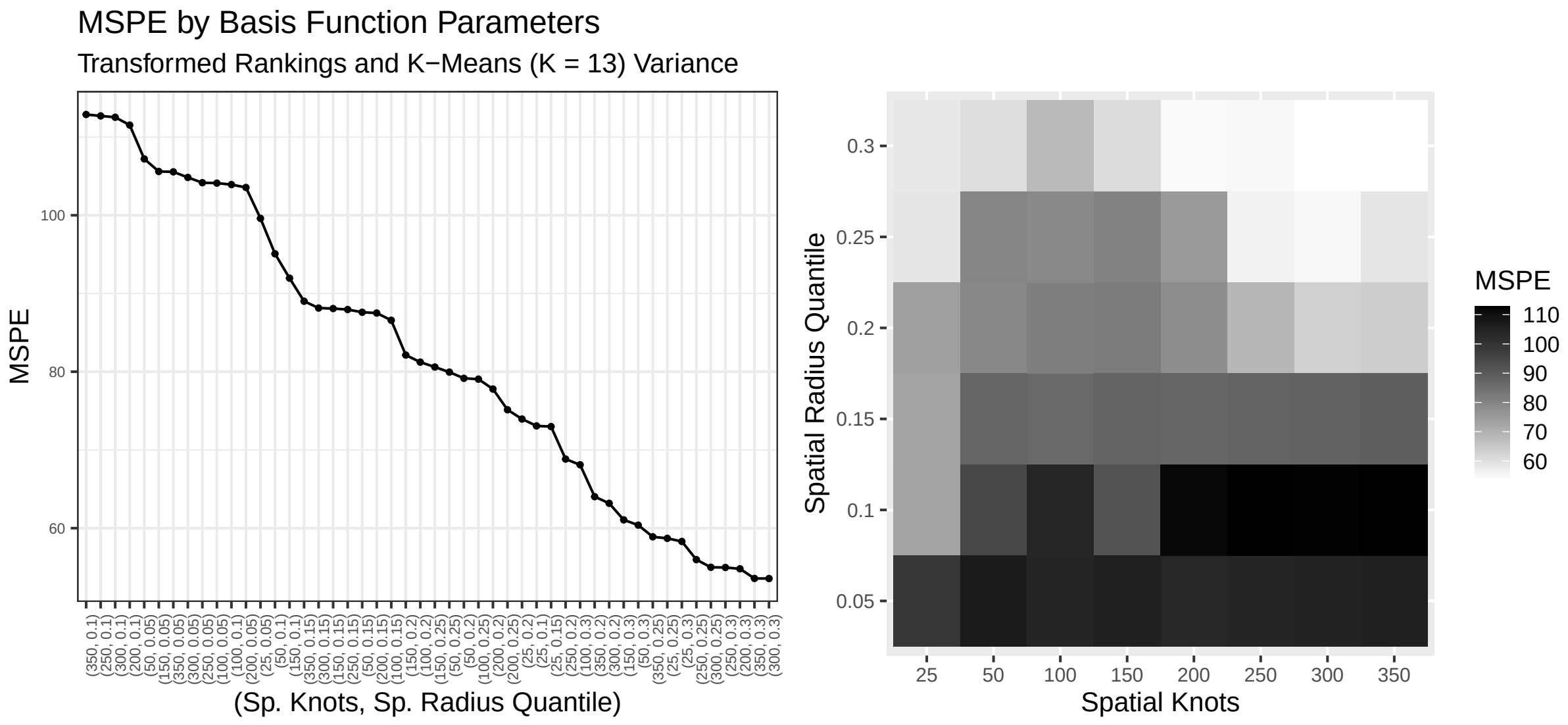

Figure 13: MSPEs by basis function parameters for models using transformed ADI rankings, the K-Means (K = 13) variance estimate method, and the S method for the computation of K.

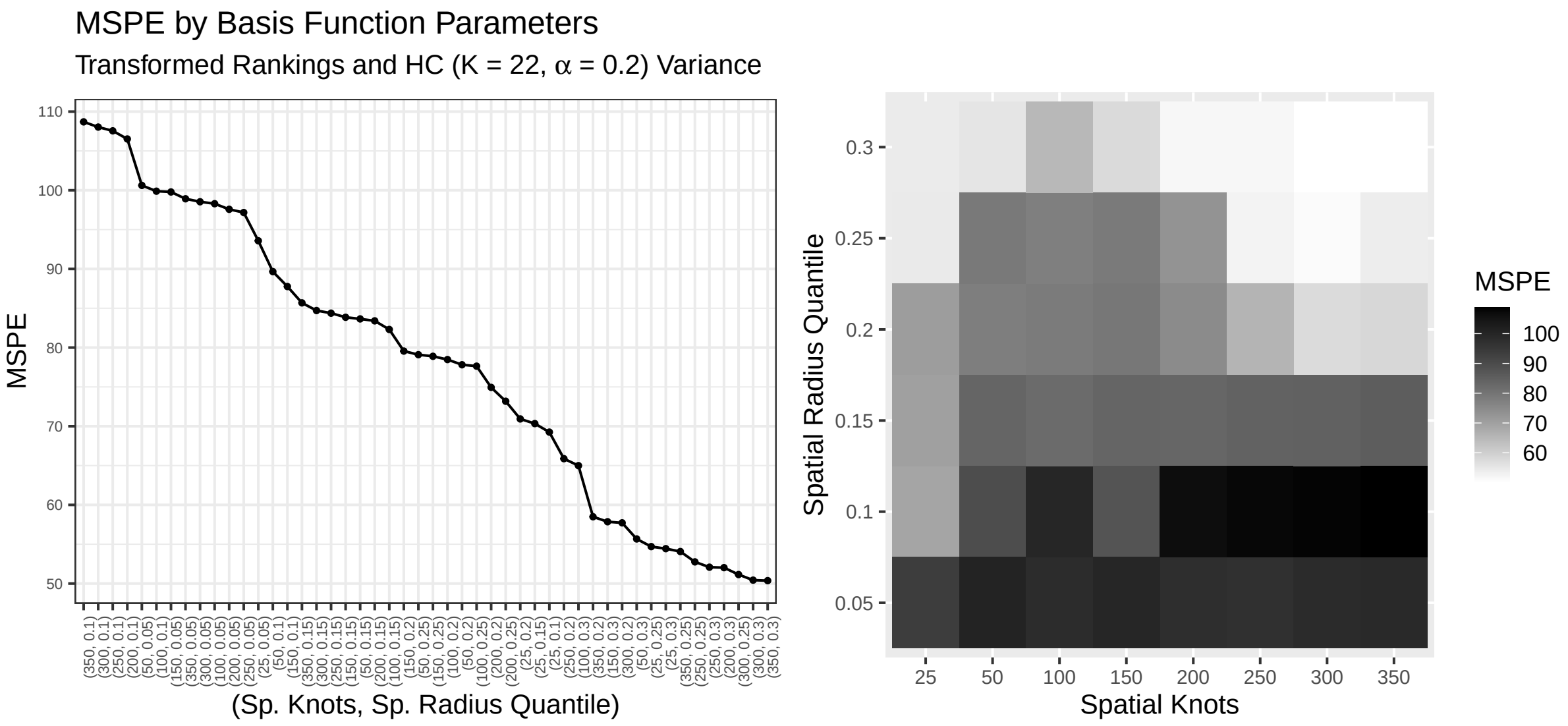

Figure 14: MSPEs by basis function parameters for models using transformed ADI rankings, the HC (K = 22, α = 0.2) variance estimate method, and the S method for the computation of K.

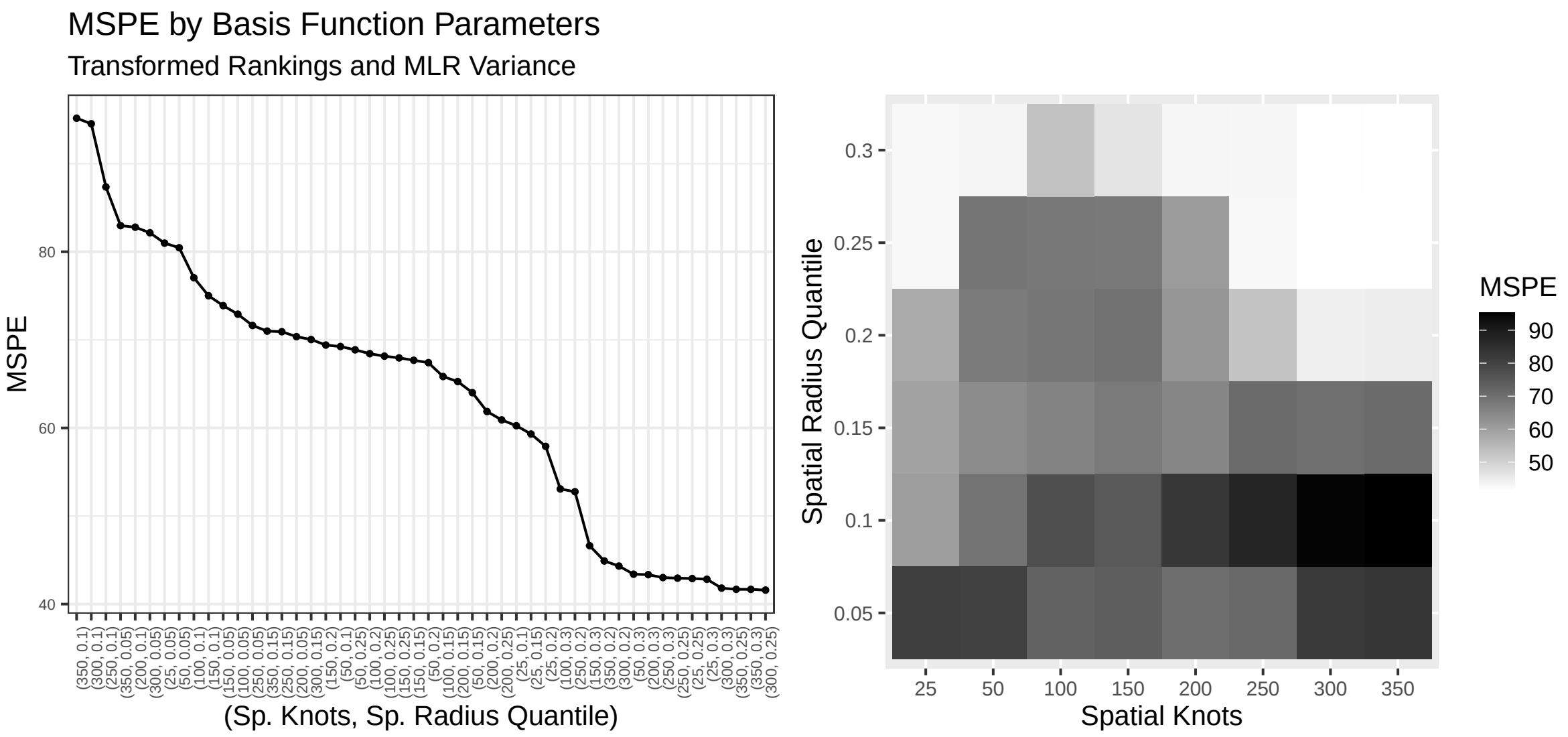

Figure 15: MSPEs by basis function parameters for models using transformed ADI rankings, the MLR variance estimate method, and the S method for the computation of K.

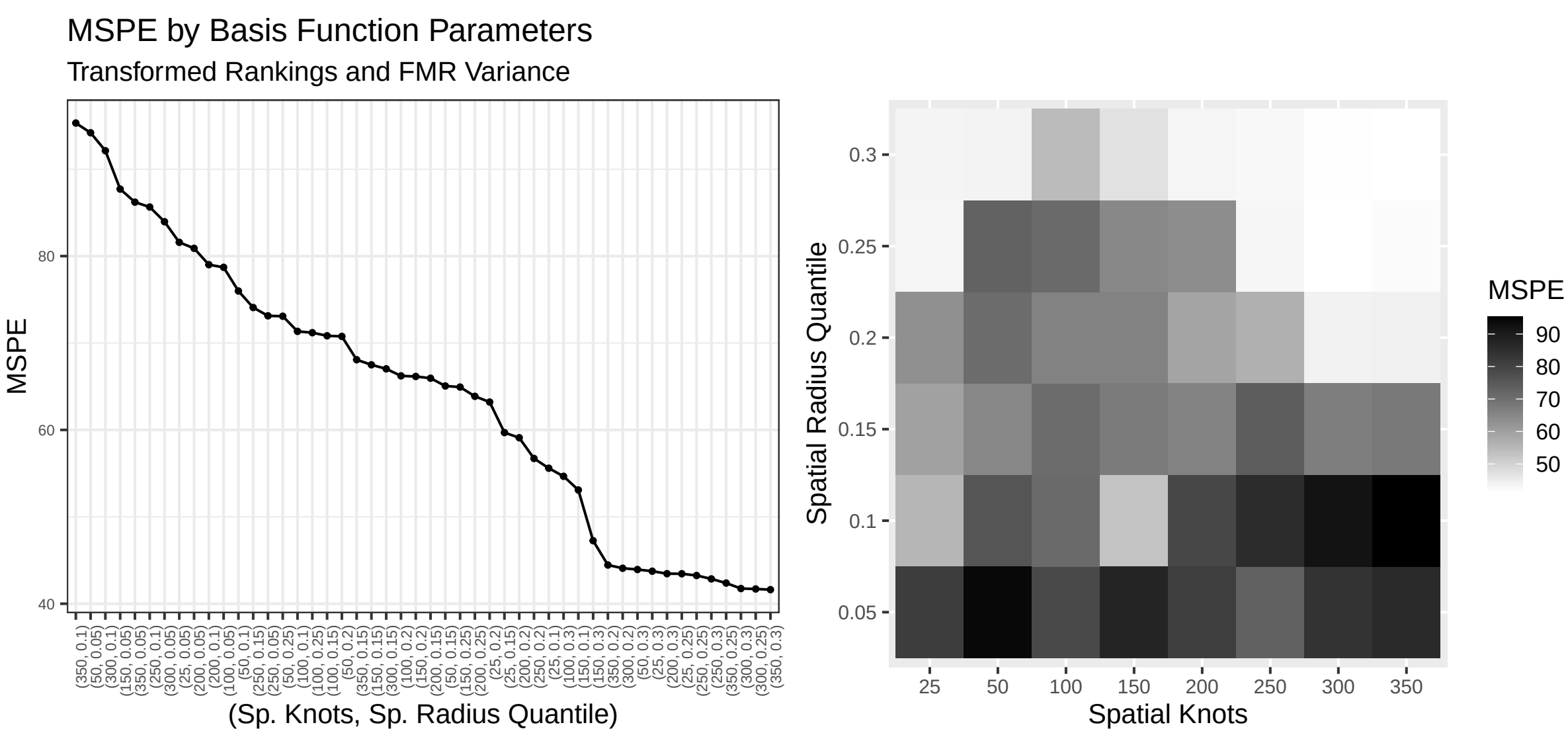

Figure 16: MSPEs by basis function parameters for models using transformed ADI rankings, the FMR variance estimate method, and the S method for the computation of K.

4 Appendix D: Model Estimates

This section provides maps of 2021 ZCTA ADI ranking model estimates and their standard deviations. Figures 17 - 21 show the estimates and standard deviations from the top models using the Yearly, KNN, K-means, HC, and FMR variance estimation methods.

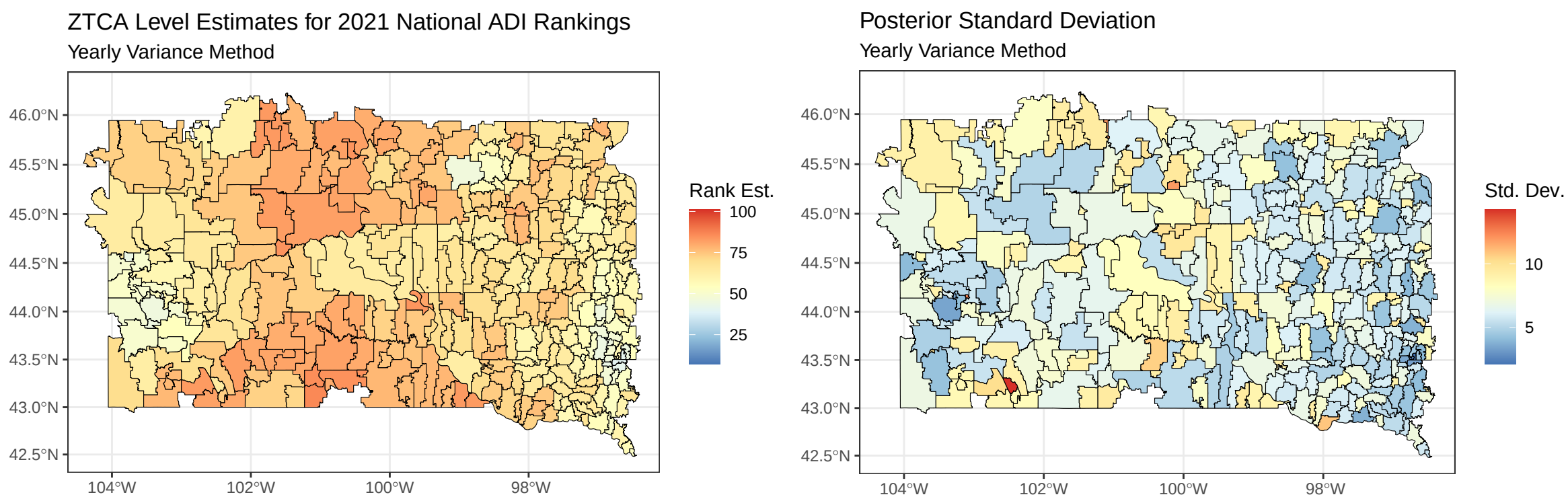

Figure 17: Yearly model estimates for 2021 South Dakota ZCTA ADI rankings and the associated posterior distributions.

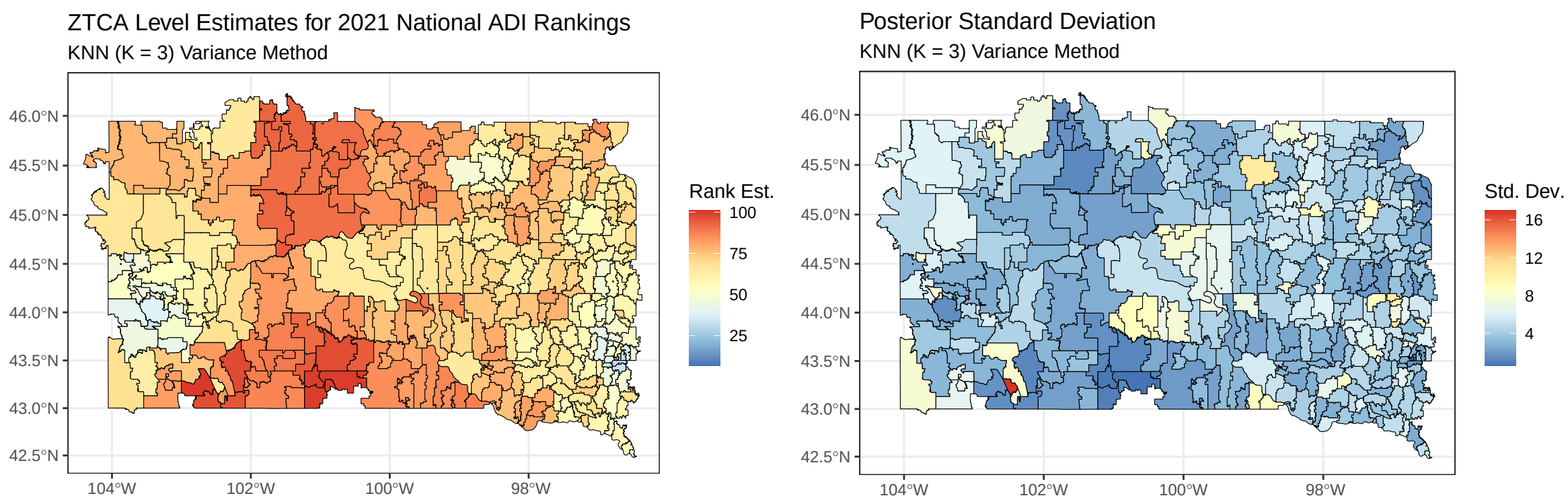

Figure 18: KNN (K = 3) model estimates for 2021 South Dakota ZCTA ADI rankings and the associated posterior standard deviations.

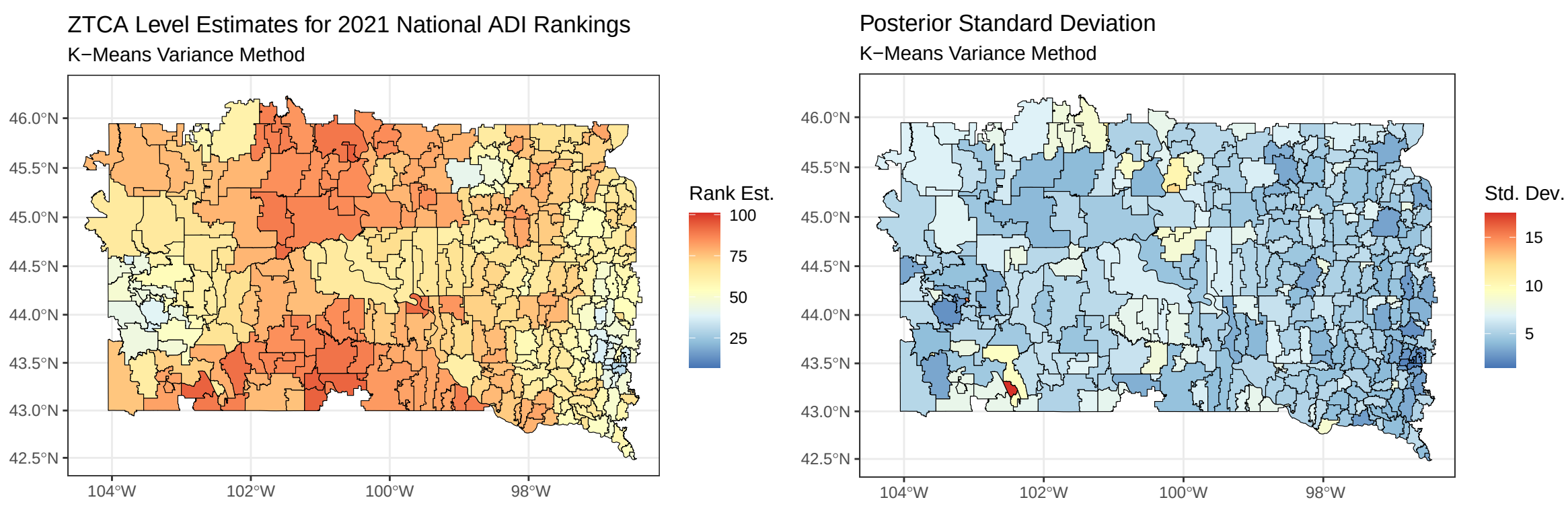

Figure 19: K-Means (K = 13) model estimates for 2021 South Dakota ZCTA ADI rankings and the associated posterior standard deviations.

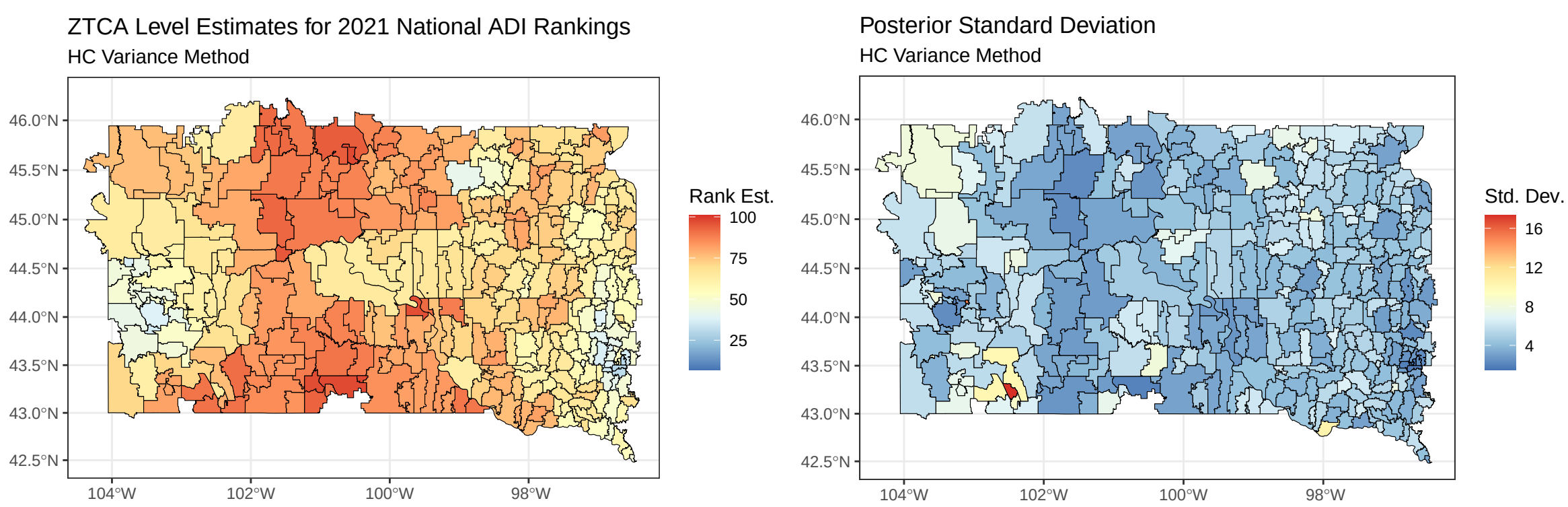

Figure 20: HC (K = 22, $\alpha = 0.2$) model estimates for 2021 South Dakota ZCTA ADI rankings and the associated posterior standard deviations.

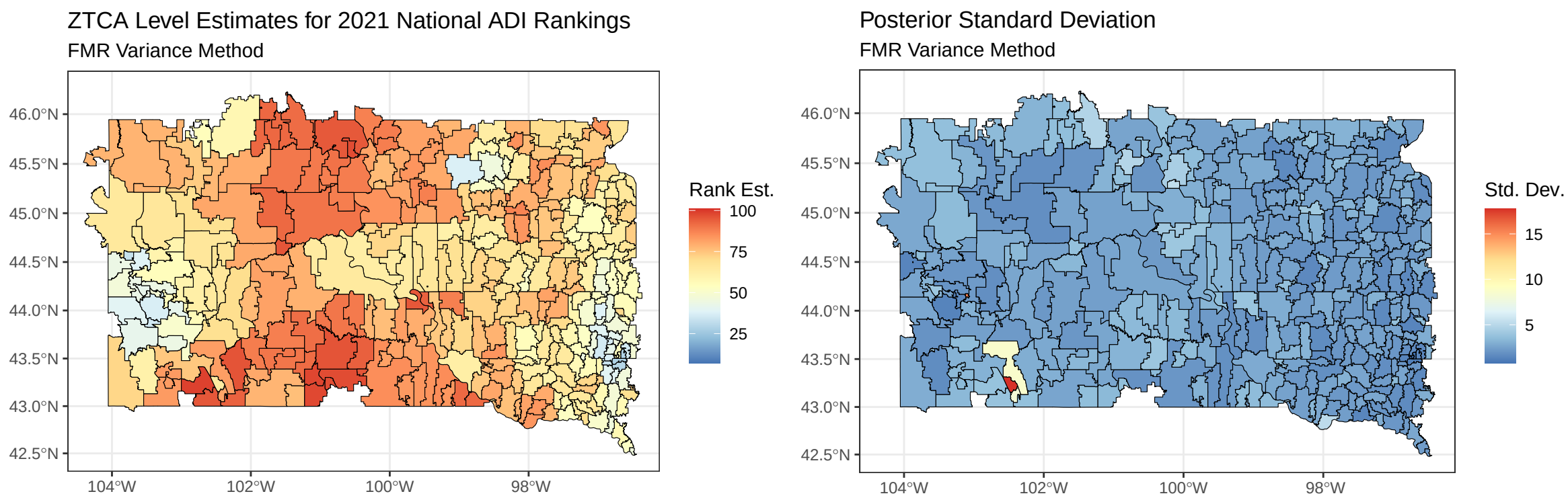

Figure 21: FMR model estimates for 2021 South Dakota ZCTA ADI rankings and the associated posterior standard deviations.

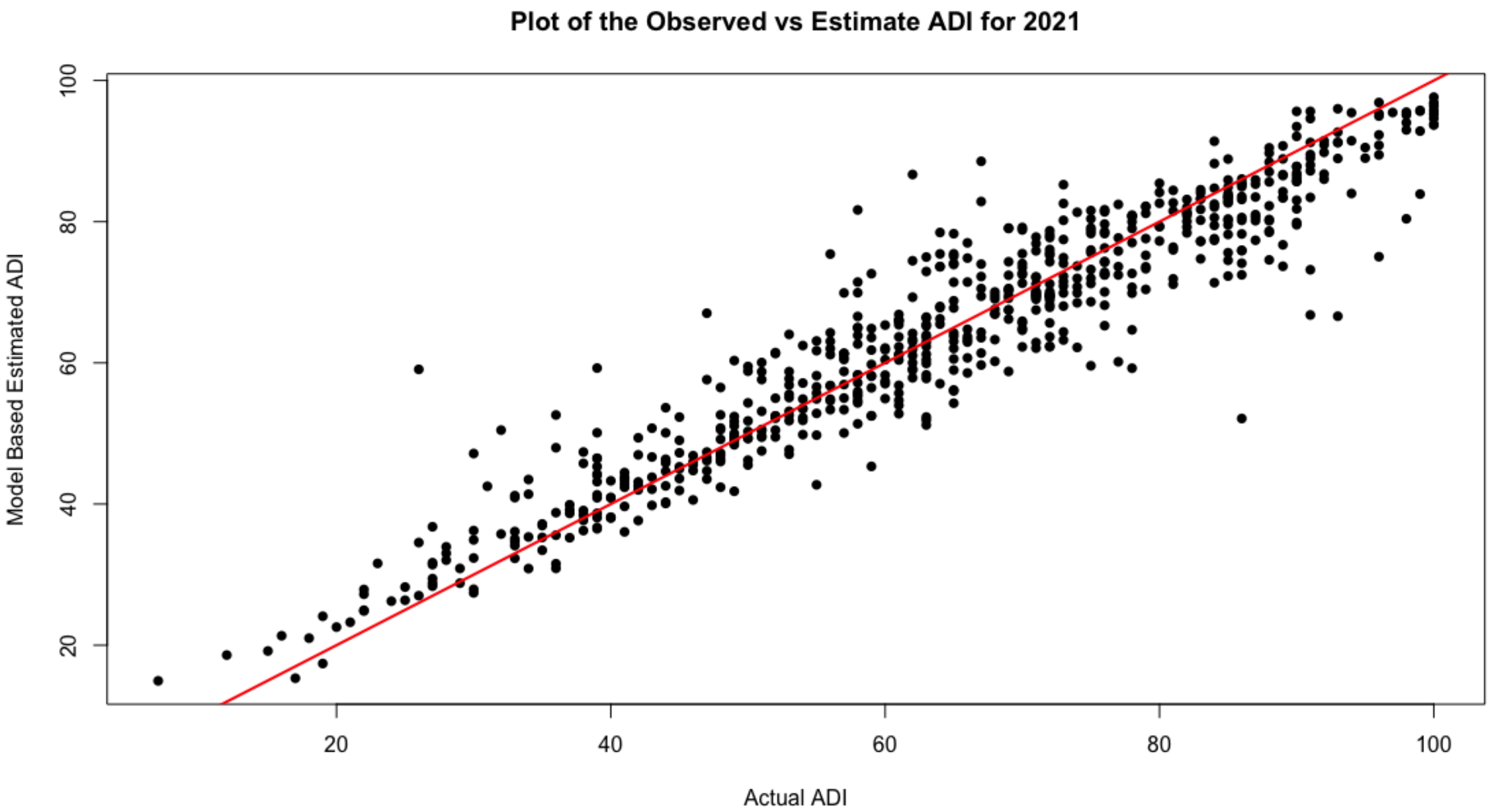

Figure 22: Plot of the observed ADI versus predicted ADI for 2021 at the census tract level. The red line shows the 45 degree line.

References

U.S. Census Bureau. Zip code tabulation areas (zctas). United States Census Bureau, 2023. Accessed via Census Bureau geographic documentation.

Nancy Krieger, Jarvis T Chen, Pamela D Waterman, Mah-Jabeen Soobader, SV Subramanian, and Rosa Carson. Geocoding and monitoring of us socioeconomic inequalities in mortality and cancer incidence: does the choice of area-based measure and geographic level matter? the public health disparities geocoding project. *American journal of epidemiology*, 156(5):471–482, 2002.

Centers for Disease Control and Prevention. Places: Local data for better health. CDC, 2023.

Agency for Healthcare Research and Quality. Healthcare cost and utilization project (hcup). AHRQ, 2023.

National Center for Health Statistics. Vital statistics data. Centers for Disease Control and Prevention, 2023.

Centers for Disease Control and Prevention. Covid-19 case surveillance and vaccination data. CDC, 2022.

Centers for Medicare & Medicaid Services. Medicare and medicaid claims data. CMS, 2023.
